# Regulation of hematogenous tumor metastasis by acid sphingomyelinase

**DOI:** 10.15252/emmm.201404571

**Published:** 2015-04-07

**Authors:** Alexander Carpinteiro, Katrin Anne Becker, Lukasz Japtok, Gabriele Hessler, Simone Keitsch, Miroslava Požgajovà, Kurt W Schmid, Constantin Adams, Stefan Müller, Burkhard Kleuser, Michael J Edwards, Heike Grassmé, Iris Helfrich, Erich Gulbins

**Affiliations:** 1 Department of Molecular Biology, University of Duisburg-EssenEssen, Germany; 2 Department of Hematology, University of Duisburg-EssenEssen, Germany; 3 Institute for Nutritional Science, University of PotsdamNuthetal, Germany; 4 Department of Genetics and Breeding Biology, Slovak University of AgricultureNitra, Slovakia; 5 Department of Pathology and Neuropathology, University of Duisburg-EssenEssen, Germany; 6 Department of Nuclear Medicine, University of Duisburg-EssenEssen, Germany; 7 Department of Surgery, University of CincinnatiCincinnati, OH, USA; 8 Department of Dermatology, University of Duisburg-EssenEssen, Germany

**Keywords:** acid sphingomyelinase, ceramide, integrins, platelets, tumor-metastasis

## Abstract

Metastatic dissemination of cancer cells is the ultimate hallmark of malignancy and accounts for approximately 90% of human cancer deaths. We investigated the role of acid sphingomyelinase (Asm) in the hematogenous metastasis of melanoma cells. Intravenous injection of B16F10 melanoma cells into wild-type mice resulted in multiple lung metastases, while Asm-deficient mice (*Smpd1*^−/−^ mice) were protected from pulmonary tumor spread. Transplanting wild-type platelets into Asm-deficient mice reinstated tumor metastasis. Likewise, Asm-deficient mice were protected from hematogenous MT/*ret* melanoma metastasis to the spleen in a mouse model of spontaneous tumor metastasis. Human and mouse melanoma cells triggered activation and release of platelet secretory Asm, in turn leading to ceramide formation, clustering, and activation of α5β1 integrins on melanoma cells finally leading to adhesion of the tumor cells. Clustering of integrins by applying purified Asm or C_16_ ceramide to B16F10 melanoma cells before intravenous injection restored trapping of tumor cells in the lung in Asm-deficient mice. This effect was revertable by arginine-glycine-aspartic acid peptides, which are known inhibitors of integrins, and by antibodies neutralizing β1 integrins. These findings indicate that melanoma cells employ platelet-derived Asm for adhesion and metastasis.

See also: YA Hannun & B Newcomb (June 2015)

## Introduction

Metastasis of tumors is one of the most important clinical problems in medical oncology. Tumor metastasis often determines a patient's prognosis, and, at present, the prognosis of patients with metastasized tumors is in general very poor. It is therefore of outstanding importance to further elucidate the molecular mechanisms of tumor metastasis.

Hematogenous metastasis of tumor cells is a multi-step process that requires the invasion of tumor cells into blood vessels; the interaction of these tumor cells with platelets, leukocytes, endothelial cells, and components of the extracellular matrix; and finally the migration of the tumor cells from the blood vessel into a distant parenchyma (Mehlen & Puisieux, 2006). Once a neoplastic cell has invaded the host's circulatory system, it must survive in a hostile environment that includes the potential for mechanical damage, the absence of growth factors from the cell's original environment, and the host's immune system. Most circulating tumor cells do not survive this stage (Liotta, 1992). Natural killer (NK) cells provide the most effective activity against tumor cells in the blood stream, at least in mice, and the depletion of NK cells results in strongly increased metastasis (Wiltrout *et al*, 1985; Nieswandt *et al*, 1999).

Studies on mice revealed that tumor cells in the blood stream interact directly with platelets and that the depletion of platelets or the inhibition of their function leads to a decrease in the number of metastases (Gasic *et al*, 1968, 1973; Crissman *et al*, 1988). Furthermore, it has been reported that the capability of murine and human tumor cells to induce platelet aggregation *in vitro* correlates with the metastatic potential of these cells *in vivo* (Honn *et al*, 1992). Platelets may contribute to metastasis by accumulating on embolic tumor cells and thus protecting them from clearance by the immune system (Nieswandt *et al*, 1999). In addition, platelets may promote metastasis by facilitating tumor cell trapping and adhesion to the endothelium (Kim *et al*, 1998). However, although the formation of tumor cell–platelet interactions is well recognized, the detailed mechanisms leading to these interactions remain unknown.

In addition to the interaction of tumor cells with platelets, the direct or indirect interaction of tumor cells with endothelial cells and with components of the extracellular matrix via adhesive receptors is essential for tumor metastasis (Humphries *et al*, 1986; Bretti *et al*, 1989; Kramer *et al*, 1989; Ruoslahti & Giancotti, 1989). Many of these adhesion receptors have been identified as members of the integrin family (Ruoslahti & Giancotti, 1989). Integrins are composed of two subunits, α and β, which are non-covalently associated with each other (Giancotti & Ruoslahti, 1999; Hynes, 2002). To date, 18 α subunits and 8 β subunits have been described, and these subunits heterodimerize into a total of 24 integrin receptors. Integrins bind to specific components of the extracellular matrix (ECM) or to respective cellular counter-receptors (Hynes, 2002). Integrins signal through the cell in either direction: the extracellular binding activity of integrins is regulated from within the cell (inside-out signaling), whereas the binding to the ECM or the respective cellular counter-receptor elicits signals that are transmitted into the cell (outside-in signaling) (Hynes, 2002). These signals regulate cell survival, the control of transcription, cell proliferation, cell motility, and cytoskeletal organization.

The expression and activity of various integrins by tumor cells is different from their expression by normal tissue cells (Nip *et al*, 1992; Pignatelli *et al*, 1992; Yoshihara *et al*, 1992). Although it is not clear whether the switch of integrin expression and activity in primary tumor cells is a consequence or a cause of malignant transformation, several experimental findings corroborate the hypothesis that certain integrins influence metastatic behavior. For instance, it has been demonstrated that blocking α5β1 integrins on B16F10 melanoma cells dramatically decreases the number of metastases (Qian *et al*, 2005).

At present, the role of acid sphingomyelinase (human: ASM, mouse: Asm) and ceramide in hematogenous tumor metastasis is poorly defined. Ceramide is formed by the hydrolysis of sphingomyelin, by the degradation of complex sphingolipids, by reverse ceramidase activity, or by *de novo* synthesis (Schuchman *et al*, 1991; Okino *et al*, 2003; Ishibashi *et al*, 2007).

Acid, neutral, and alkaline sphingomyelinases, which exhibit their peak activity at specific pH values, catalyze the hydrolysis of sphingomyelin (Kolesnick *et al*, 2000; Hannun & Obeid, 2008). Ceramide and Asm have been shown to play a pivotal role in the signal transduction of many stress and apoptotic stimuli, including in platelets (Schissel *et al*, 1996, 1998a; Simon *et al*, 1998; Romiti *et al*, 2000; Grassmé *et al*, 2001; Jin *et al*, 2008). A comprehensive model explaining the role of Asm-released ceramide in these diverse systems indicates that ceramide generated by the activity of Asm results in the reorganization of the membrane and the formation of ceramide-enriched membrane platforms (Grassmé *et al*, 2001). Ceramide-enriched membrane platforms are generated by the unique properties of ceramide molecules that spontaneously associate with each other and separate from other phospholipids in the cell membrane to form distinct domains. These ceramide-enriched membrane domains serve to reorganize receptor molecules and signaling molecules, that is, to cluster activated receptors and to trap and enrich intracellular signaling molecules at the site of receptor clusters (Grassmé *et al*, 2001). This reorganization of receptors and associated signaling molecules amplifies initial signals and permits the transmission of signals into the cell.

In this study, we demonstrate that the interaction of tumor cells with platelets results in the secretion of Asm from platelets and that this secreted Asm, in turn, mediates the formation of ceramide-enriched membrane platforms on tumor cells. These platforms serve to cluster integrins, a process that is required for tumor metastasis into the lung. Genetic and pharmacological inhibition of the Asm prevents tumor metastasis.

## Results

### Expression of Asm is required for pulmonary metastasis

**Figure 1:**
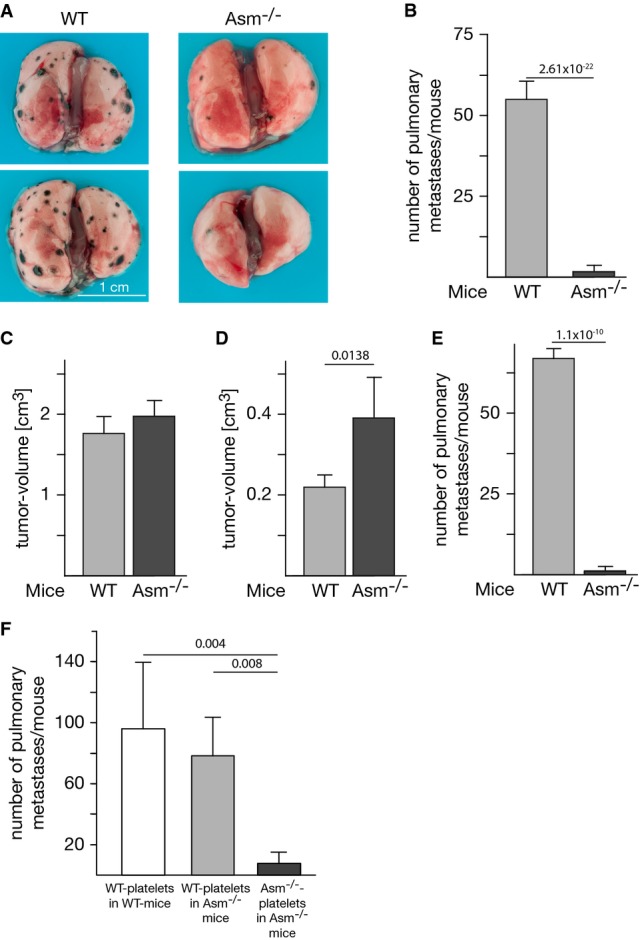
Tumor metastasis is abrogated in Asm-deficient mice and restored by transplantation of wild-type platelets

To define the role of Asm in hematogenous tumor metastasis, we intravenously injected B16F10 melanoma cells into C57BL/6 mice; this injection resulted in multiple lung metastases in these mice after 14 days (Fig1A and B). In marked contrast, tumor metastasis was almost completely absent after B16F10 melanoma cells were injected into syngeneic Asm-deficient mice (*Smpd1*^−/−^ mice) (Fig1A and B). This finding indicates that the expression of Asm is required for pulmonary metastasis of B16F10 melanoma. Controls show that B16F10 melanoma cells grow as fast or even slightly faster in Asm-deficient mice than in wild-type mice after subcutaneous injection at the flank, excluding a general growth defect of the tumor in Asm-deficient mice (Fig1C). To further prove that the local growth of the tumor in the lung is not affected by Asm deficiency, we injected B16F10 melanoma cells directly into the lung and determined tumor size compared to wild-type mice (Fig1D). Further, even if metastasis in Asm-deficient mice was analyzed after an additional 10 days (total 25 days after injection of the tumor cells), the number of metastases remained low (Fig1E), excluding that small metastases were not detected at the analysis of tumors 14 days after i.v. injection.

A, B 1 × 10^5^ B16F10 melanoma cells were intravenously injected into C57BL/6 wild-type (WT) or Asm-deficient (Asm^−/−^) mice, and the number of lung metastases was determined after 14 days. The photographs (A) show two representative results. The graph (B) shows the mean ± SD number of pulmonary metastases in 15 WT and 14 Asm-deficient animals.

C, D B16F10 melanoma grow as fast or slightly faster in Asm-deficient mice than in wild-type mice after subcutaneous injection at the flank (C) or transcutaneous direct intrapulmonary injection (D), excluding a general growth defect of the tumor in Asm-deficient mice. The size of tumors was determined 14 days after local injection at the flank or 10 days after injection into the lung.

E The number of lung metastases 25 days after intravenous injection does not differ from the number observed after 14 days in (B).

F 5 × 10^8^ platelets isolated from wild-type (WT) or Asm-deficient (Asm^−/−^) mice were injected intravenously into Asm-deficient or wild-type mice, respectively. After 120 min 1 × 10^5^, B16F10 tumor cells were intravenously injected. The number of lung metastases was determined after 14 days. Displayed are the mean ± SD of the number of pulmonary metastases, *n* = 3 in WT platelets in WT mice, *n* = 5 in WT platelets in Asm^−/−^ mice, and *n* = 4 in Asm^−/−^ platelets in Asm^−/−^ mice.

Data information: Shown is the mean ± SD, *n* = 5 (C–E). Statistical significance was determined by *t*-test for single comparisons or analysis of variance (ANOVA) followed by a Tukey's multiple comparisons test. *P*-values are given.

### Transplantation of platelets prior to injection of tumor cells restores pulmonary metastasis in Asm-deficient mice

To obtain mechanistic insights into Asm-mediated tumor metastasis, we transfused Asm-deficient mice with wild-type platelets. This procedure restored the generation of lung metastases by B16F10 cells (Fig1F), an outcome demonstrating that platelet-derived Asm mediates the observed role of Asm in hematogenous tumor metastasis. To assess the proportion of wild-type platelets in transfused Asm-deficient mice at the time point of tumor cell injection, we injected 5 × 10^8^ platelets isolated from wild-type mice in Asm-deficient mice. Experiments were done in triplicate. After 120 min, we collected blood and isolated the platelets. We measured the Asm activity in the platelets from transfused mice and from the same number of platelets from wild-type mice and from Asm-deficient mice. From the obtained values, we calculated the proportion of wild-type and Asm-deficient platelets in transfused mice. We found that the ratio of wild-type:Asm-deficient platelets was 45:55 after transfusion of Asm-deficient mice with 5 × 10^8^ wild-type platelets.

### Interaction of tumor cells with platelets results in the activation and release of secretory Asm and in the concomitant formation of surface ceramide

**Figure 2:**
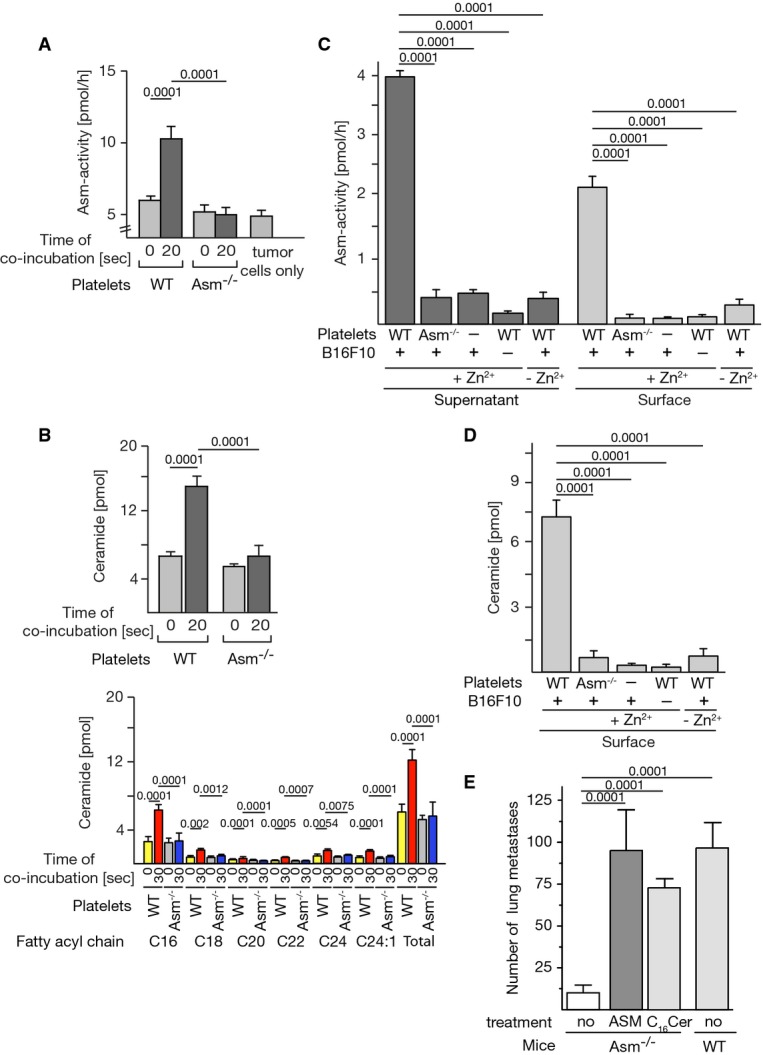
Co-incubation of B16F10 melanoma with platelets results in Asm secretion and activation and ceramide release to mediate metastasis

To elucidate the molecular mechanisms whereby Asm mediates tumor metastasis, we tested whether Asm is activated by the interaction between B16F10 melanoma cells and platelets. We found that co-incubation of wild-type platelets with B16F10 cells resulted in very rapid stimulation of Asm (Fig2A) and a concomitant formation of ceramide, as determined by kinase assay (Fig2B upper panel) and mass spectrometry (Fig2B lower panel). In contrast, no change in Asm activity or ceramide levels occurred after tumor cells were incubated with Asm-deficient platelets, a finding indicating that Asm is activated in platelets but not in tumor cells.

A Platelets were isolated from wild-type (WT) or Asm-deficient (Asm^−/−^) mice by density-gradient centrifugation, and 1 × 10^7^ platelets were incubated with 1 × 10^5^ B16F10 cells. The samples were lysed, and Asm activity was determined. In unstimulated samples (time point of co-incubation 0 s), tumor cells and platelets were admixed after lysis.

B The formation of ceramide was determined by a diacylglycerol (DAG) kinase assay (upper panel) and mass spectrometry (lower panel). In unstimulated samples (time point of co-incubation 0 s), tumor cells and platelets were admixed after lysis.

C, D For determination of the secretion of Asm by platelets into the supernatants (C), tumor cells and wild-type (WT) or Asm-deficient (Asm^−/−^) platelets were co-incubated for 20 s, the samples were pelleted, the supernatants were removed and acidified, and the Asm activity was measured in the presence or absence of Zn^2+^. For measurement of surface Asm and ceramide, samples were co-incubated as indicated (C, D) and pelleted; the supernatants were discarded, incubated with anti-Asm antibodies, washed, and lysed; and Asm immunocomplexes were immobilized and subjected to immunocomplex enzyme assays. Surface ceramide was measured by incubation of intact cells with DAG kinase in the presence of [^32^P]γATP followed by extraction and measurement of [^32^P]-ceramide. Omission of one cell type indicated by “−” here and thereafter.

E Treatment of B16F10 tumor cells for 2 min with 1 U/ml ASM or 10 μM C_16_ ceramide restores *in vivo* metastasis in Asm-deficient mice. After treatment, the cells were injected intravenously into Asm-deficient (Asm^−/−^) mice. Controls were left untreated prior to injection. The number of metastases was determined 14 days after tumor cell injection.

Data information: Displayed is the mean ± SD of 4 (A–D) or 9 (E) experiments. Statistical significance was determined by analysis of variance (ANOVA) followed by a Tukey's multiple comparisons test. *P*-values are given.

Asm resides in intracellular vesicles, in particular in lysosomes and secretory lysosomes (Jin *et al*, 2008). The fusion of secretory lysosomes results in the exposure of the membrane-bound enzyme on the outer leaflet of the cell membrane (Grassmé *et al*, 2001; Jin *et al*, 2008). Alternatively, the Asm is secreted by many cell types (Romiti *et al*, 2000; Schissel *et al*, 1996; 1998; Simon *et al*, 1998), for instance, after treatment of platelets with thrombin (Simon *et al*, 1998; Romiti *et al*, 2000). Secreted Asm then binds back to the cell surface to hydrolyze sphingomyelin. The two isoforms can be easily discriminated, since secretory, but not lysosomal, Asm depends on Zn^2+^ (Schissel *et al*, 1998a,b).

Thus, to elucidate whether co-incubation of platelets with tumor cells triggers the secretion of Asm, we measured Asm activity in the supernatant upon co-incubation of platelets with tumor cells and directly on the cell surface. In addition, we analyzed the Zn^2+^-dependency of the Asm in the supernatant and on the cell surface. We found that Asm is released from wild-type platelets into the supernatant, whereas Asm secretion is lacking when Asm-deficient platelets are co-incubated with tumor cells (Fig2C). Asm activity required addition of Zn^2+^, indicating that it is the secretory isoform of the Asm, which is released upon co-incubation of tumor cells with platelets (Fig2C).

To further define the local activity of the Asm and the local formation of ceramide, we co-incubated tumor cells with wild-type or Asm-deficient platelets, washed the cells, and used the cell pellets for immunoprecipitation of the Asm from cell surfaces or measurements of surface ceramide by performing an *in situ* ceramide kinase assay on intact cells (Fig2C and D). These data indicate that co-incubation of B16F10 cells with wild-type platelets results in surface activity of Zn^2+^-dependent Asm and the formation of surface ceramide, while neither significant surface Asm nor ceramide was detected after incubation of B16F10 tumor cells with Asm-deficient platelets. If platelet-secreted Asm is relevant for tumor cell metastasis, the *ex vivo* treatment of B16F10 melanoma cells with purified ASM should be sufficient to restore metastasis in Asm-deficient mice. To test this hypothesis, we treated B16F10 melanoma cells with 1 U/ml purified ASM *ex vivo*, washed the cells, and injected them intravenously into Asm-deficient mice. We found that the injection of tumor cells treated *ex vivo* with purified ASM restored tumor metastasis in Asm-deficient mice (Fig2E). Likewise, treatment of B16F10 melanoma cells with 10 μM C_16_ ceramide *ex vivo* restored metastasis in Asm-deficient mice (Fig2E). This finding suggests that the generation of ceramide on tumor cells is sufficient to mediate tumor cell metastasis and to bypass Asm deficiency.

**Figure 3:**
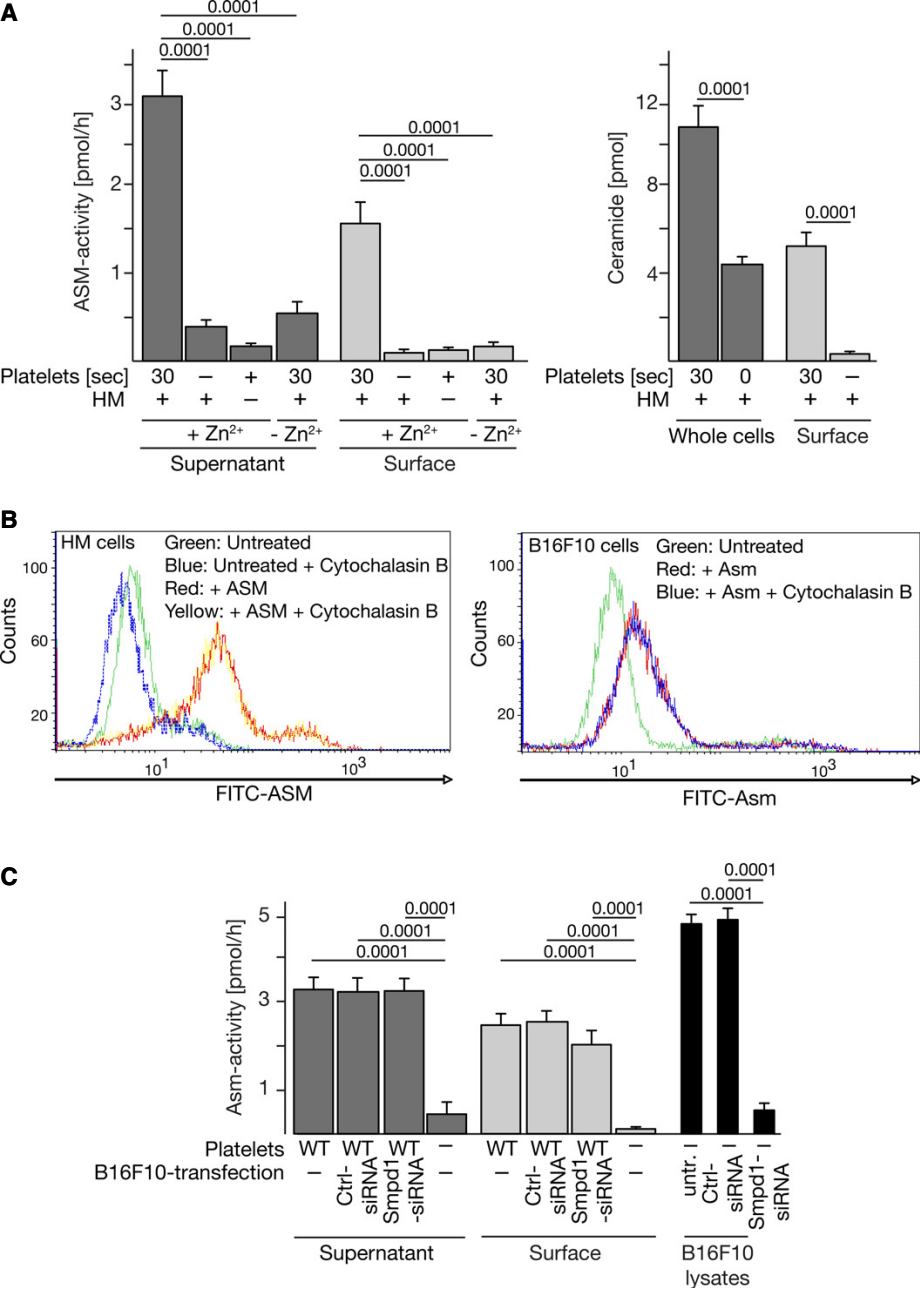
Interaction of human or mouse melanoma cells with platelets results in Asm secretion and surface Asm activity independent of Asm expression in melanoma cells

Similar data were obtained for human melanoma cells: Incubation of these cells with human platelets resulted in the formation of ceramide, the release of Zn^2+^-dependent ASM into the supernatant, and Zn^2+^-dependent activity of ASM on cell surfaces as well as the formation of surface ceramide (Fig3A).

Incubation of human melanoma (HM) cells with human platelets results in the release of Zn^2+^-dependent ASM into the supernatant, Zn^2+^-dependent activity of ASM on cell surfaces, and the formation of ceramide. The assay buffer contained 100 μM Zn^2+^.

Addition of human or mouse recombinant ASM to human melanoma or B16F10 cells, respectively, results in binding of ASM to the tumor cell surfaces as determined by flow cytometry. Cytochalasin B was added to control for internalization of added ASM.

Suppression of Asm in B16F10 tumor cells using siRNA technology reduces Asm activity in B16F10 cells (right panel), but does not alter release or surface activity of the Asm after co-incubation with wild-type platelets (left panels).

Data information: In (A) and (C) are shown the mean ± SD, *n* = 4. In (B) representative data from four independent experiments each are displayed. Statistical significance was determined using ANOVA followed by a Tukey's multiple comparisons test. *P*-values are indicated (Ctrl: control).

Addition of human or mouse recombinant ASM/Asm to human melanoma or B16F10 cells, respectively, resulted in binding of the ASM/Asm to the tumor cell surfaces (Fig3B) as determined by FACS analysis.

To further prove that Asm originates from platelets after co-incubation with B16F10 tumor cells, we suppressed Asm in B16F10 tumor cells using siRNA technology. Suppression was 90% as determined by enzymatic activity measurements (Fig3C right). The siRNA-mediated suppression did not alter release of the acid sphingomyelinase after co-incubation of tumor cells with wild-type platelets (Fig3C left). It also did not affect the activity of surface acid sphingomyelinase as determined by immunocomplex assays of surface Asm (Fig3C middle). These studies clearly demonstrate that the Asm on the surface is derived from platelets upon co-incubation of both cell types.

### Activation of platelets by B16F10 melanoma cells is not impaired by Asm deficiency

**Figure 4:**
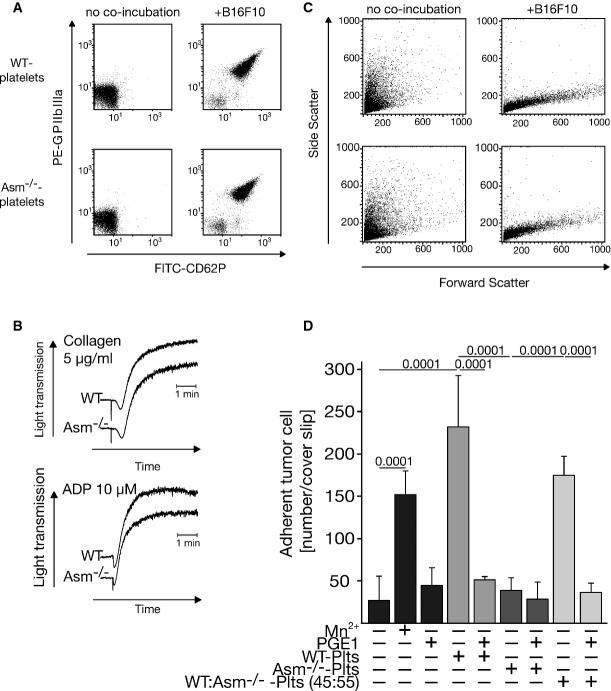
The primary interaction of tumor cells and platelets is not affected by Asm deficiency, but Asm expression in platelets is required for tumor cell adhesion

To exclude the possibility that the failure of Asm-deficient platelets to respond to B16F10 tumor cells is caused by the absence of an interaction between tumor cells and platelets, we performed control experiments that determined typical markers of very early activation of platelets, such as the upregulation of CD62P and GPIIbIIIa. We found no difference between wild-type and Asm-deficient platelets in the upregulation of these early platelet activation markers upon the interaction of tumor cells and platelets (Fig4A). Additional studies demonstrated that the aggregation of platelets upon stimulation with platelet agonists such as adenosine diphosphate (ADP), collagen, collagen-related peptide (CRP), and U46619 was not altered in Asm-deficient platelets (Fig4B and not shown). Further FACS analyses demonstrated degranulation and change of shape of platelets after stimulation with B16F10 melanoma cells (Fig4C). Thus, a general defect in platelet function or in the interaction of platelets with tumor cells cannot be responsible for the inhibition of tumor metastasis in Asm-deficient mice.

Platelets were isolated from wild-type (WT) or Asm-deficient (Asm^−/−^) mice by density-gradient centrifugation, and 1 × 10^7^ platelets were incubated with 1 × 10^5^ B16F10 cells or left untreated. Co-incubation of washed platelets with B16F10 melanoma cells resulted in upregulation of GPIIbIIIa and CD62P on both wild-type and Asm-deficient platelets in FACS analyses. Platelets were gated in FCS/SSC.

Aggregation properties of platelets after co-incubation with collagen or ADP are independent of Asm as determined by aggregometry measurements.

Incubation of tumor cells with platelets resulted in marked change of platelet shape and degranulation as determined by FACS analysis using forward versus side scatter analysis indicating global activation of platelets.

4 × 10^4^ B16F10 tumor cells were incubated with 2 × 10^7^ wild-type (WT), Asm-deficient (Asm^−/−^) platelets (Plts) or a 45:55 of WT:Asm^−/−^ platelet mixture in the presence or absence of 50 ng/ml PGE1. Controls were stimulated with Mn^2+^. Tumor cells were then incubated for 60 s on fibronectin-coated cover slips, washed, and fixed, and adhesion of the tumor cells was determined. The graph displays the mean ± SD of tumor cells adhering to fibronectin-coated cover slips, *n* = 7 for WT-platelets, *n* = 9 for Asm-deficient-platelets, *n* = 4 for 45:55 WT:Asm^−/−^ platelets, and all others *n* = 3. Statistical significance was determined by analysis of variance (ANOVA) followed by Tukey's multiple comparisons test. *P*-values are indicated.

Data information: Displayed are representative results of each four independent experiments (A–C).

Collectively, these findings indicate that the contact between tumor cells and platelets results in the activation and secretion of platelet-derived Asm; this activity is important for metastasis.

### Wild-type platelets, but not Asm-deficient platelets, promote tumor cell adhesion *in vitro*

To gain insights into the mechanisms of Asm-mediated metastasis of B16F10 melanoma cells, we determined whether platelet-derived Asm regulates the adhesion of tumor cells. Adhesion factors such as integrins have been previously shown to be crucial for tumor metastasis (Bretti *et al*, 1989; Juliano & Varner, 1993; Qian *et al*, 2005). We incubated B16F10 melanoma cells with wild-type or Asm-deficient platelets and determined the adhesion of these cells to fibronectin-coated glass cover slips. While spontaneous adhesion of untreated B16F10 cells was low, we observed a very rapid adhesion of tumor cells upon co-incubation with wild-type platelets. In contrast, Asm-deficient platelets failed to promote B16F10 cell adhesion (Fig4D). To further test the role of platelets in this process, we inhibited platelet functions by pre-incubation with PGE1. PGE1 inhibited tumor cell adhesion upon co-incubation with wild-type platelets (Fig4D). Since PGE1 is a well-known platelet inhibitor, these data support the proposed mechanism of Asm-mediated tumor cell adhesion.

To determine whether the proportion of wild-type platelets obtained in Asm-deficient mice after transfusion with wild-type platelets (in analogy to the *in vivo* experiments) is sufficient to promote adhesion, we stimulated B16F10 melanoma cells with wild-type platelets, Asm-deficient platelets, or a mixture of wild-type and Asm-deficient platelets in a ratio of 45:55. The results show that 45% wild-type platelets are able to fully restore adhesion of the tumor cells. Furthermore, stimulation of B16F10 melanoma cells with Mn^2+^ resulted in a marked increase in adhesion (Fig4D).

### Purified ASM and wild-type platelets, but not Asm-deficient platelets, induce clustering of α5 and β1 integrins in ceramide-enriched platforms, β1 integrin activation, and tumor cell adhesion

**Figure 5:**
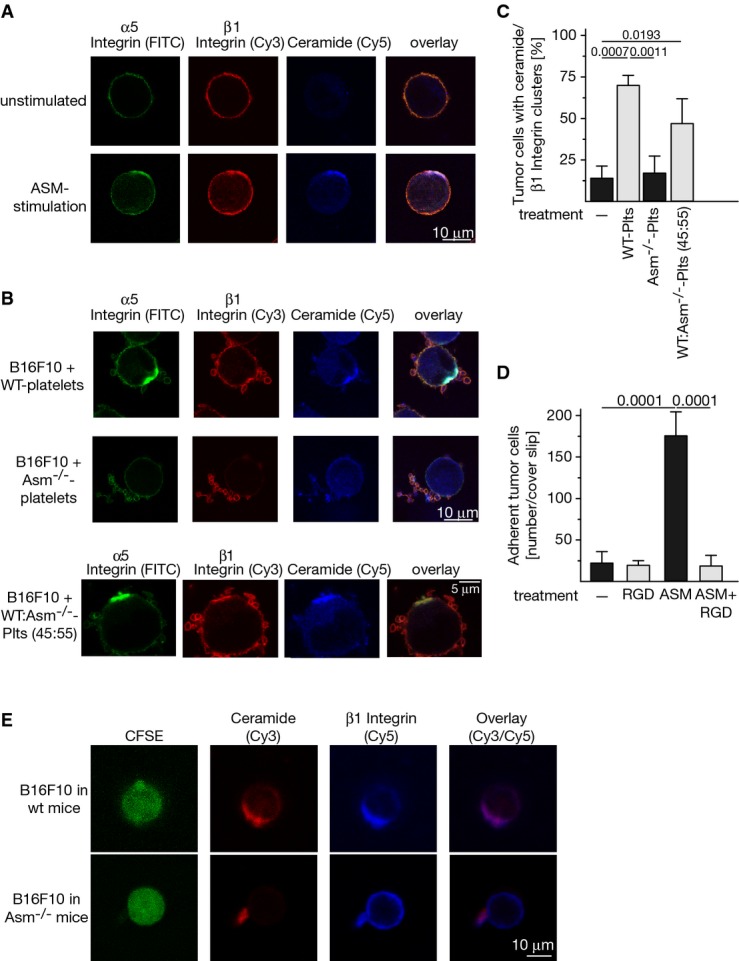
Acid sphingomyelinase induces clustering and activation of α5 and β1 integrins in ceramide-enriched membrane domains resulting in tumor cell adhesion and metastasis

Ceramide has been previously shown to trigger clustering of receptor molecules in the cell membrane; this clustering serves to initiate and amplify receptor-mediated signals (Grassmé *et al*, 2001). We found that the incubation of tumor cells with purified ASM resulted in the formation of ceramide-enriched membrane platforms on B16F10 cells and that α5 and β1 integrins clustered in these membrane platforms (Fig5A). Most important, the incubation of wild-type platelets with B16F10 tumor cells also resulted in very rapid clustering of α5 and β1 integrins in ceramide-enriched membrane platforms on the tumor cells, whereas Asm-deficient platelets had no effect (Fig5B). Furthermore, we investigated clustering of integrins in B16F10 tumor cells after incubation with a mixture of wild-type and Asm-deficient platelets in a ratio of 45:55. We found that this ratio of wild-type and Asm-deficient platelets is sufficient to mediate clustering of α5 and β1 integrins in ceramide-enriched membrane platforms in B16F10 tumor cells (Fig5B and C). Treatment of the cells with arginine-glycine-aspartic acid (RGD) peptides that block the interactions between integrins, in particular α5β1, and their ligands (Hynes, 2002), almost completely prevented the adhesion of ASM-treated tumor cells to fibronectin-coated cover slips (Fig5D).

A–C 1 × 10^5^ B16F10 cells were incubated with (A) 1 U/ml purified acid sphingomyelinase (ASM) or (B) 5 × 10^7^ wild-type (WT), Asm-deficient (Asm^−/−^) platelets (Plts), or a mixture of 45:55 wild-type:Asm-deficient platelets. Cells were fixed with 2% paraformaldehyde for 15 min and stained with fluorescein isothiocyanate (FITC)-coupled anti-α5 integrin, Cy3-labeled anti-β1 integrin, and Cy5-labeled anti-ceramide antibodies. Samples were analyzed by confocal microscopy. Shown are representative examples from four independent experiments (A, B) or the quantitative analysis of cells positive for ceramide/β1 integrin clusters from at least 100 cells/sample (C). Given is the mean ± SD, *n* = 4, ANOVA followed by Tukey's multiple comparisons test. *P*-values are indicated.

D ASM-induced B16F10 tumor cell adhesion to fibronectin-coated cover slips is abrogated by the inhibition of integrins with RGD peptides. Shown is the mean ± SD of the number of cells adherent to the cover slip. Statistical significance was determined by ANOVA followed by Tukey's multiple comparisons test. *P*-values are indicated.

E Intravenous injection of CFSE-labeled B16F10 cells into wild-type mice results in formation of ceramide-enriched domains that contain β1 integrin clusters on tumor cells *in vivo*, while injection of tumor cells into Asm-deficient mice does not result in ceramide and integrin clustering. Please note that the cell in the lower panel still binds a platelet. Shown are representative results from four independent experiments each.

To prove clustering of β1 integrin in ceramide-enriched membrane platforms *in vivo*, we labeled B16F10 tumor cells with CFSE, injected the cells into wild-type or acid sphingomyelinase-deficient mice, took blood 5 min after injection, performed a Ficoll gradient to remove erythrocytes and to purify tumor cells, fixed the cells, and stained with Cy3-anti-ceramide and Cy5-anti-β1 integrin. The results show formation of ceramide-enriched domains that contain β1 integrin cluster in tumor cells (identified by the intense green labeling of CFSE) after injection into wild-type mice, while injection into Asm-deficient mice did not result in ceramide and β1 integrin clustering at the surface of tumor cells (Fig5E). These data show that clustering of ceramide and ß1 integrin also occurs *in vivo*.

**Figure 6:**
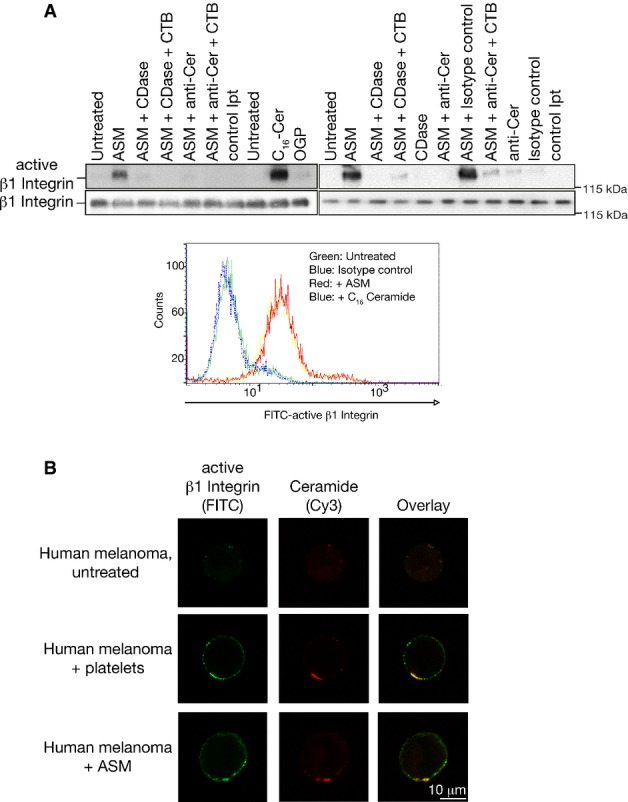
Acid sphingomyelinase induces clustering and activation of β1 integrins in ceramide-enriched membrane domains in human melanoma cells

To gain insight into the question whether ceramide activates integrin, we employed human melanoma cells, which can be analyzed with the HUTS-4 antibody that exclusively detects active β1 integrin molecules. We treated human melanoma cells with ASM, or C_16_ ceramide. These stimulations resulted in activation of β1 integrin as shown by immunoprecipitation studies using the HUTS-4 antibody (Fig6A). Activation of β1 integrin was prevented by co-incubation of the samples with ceramidase to consume ceramide or anti-ceramide antibodies to block surface ceramide (Fig6A). Activation of β1 integrin in human melanoma cells by incubation with ASM or C_16_ ceramide was confirmed by FACS analysis upon staining with FITC-labeled HUTS-4 antibodies (Fig6A).

Human melanoma cells were stimulated for 10 min with 1 U/ml purified ASM or 10 μM C_16_ ceramide (C16-Cer) in the presence or absence of 500 ng ceramidase (CDase) or 1 μg/ml neutralizing anti-ceramide antibodies (anti-Cer), and lysed and active β1 integrin was immunoprecipitated using the HUTS-4 antibody. Octylglucopyranoside (OGP, final concentration 0.01%) served to resuspend C_16_ ceramide. Internalization was excluded by addition of cytochalasin B (CTB) as indicated. Samples were separated by SDS-PAGE and blotted with an activation-independent anti-β1 Integrin antibody to detect the amount of immunoprecipitated, that is active β1 integrin. Control immunoprecipitates (control Ipt) were performed with an irrelevant isotype control antibody. Activation of β1 integrin in human melanoma cells by incubation with Asm or C_16_ ceramide was confirmed by FACS analysis upon staining with FITC-labeled HUTS-4 antibodies.

Incubation of human melanoma with human platelets or ASM for 5 min triggers co-clustering of ceramide and activated β1 integrin on the surface of the tumor cells. Cells were stained with FITC-coupled anti-active β1 integrin (HUTS-4) antibodies and Cy3-coupled anti-ceramide antibodies. Shown are representative stainings from each four independent experiments.

Source data are available online for this figure.

Finally, we incubated human melanoma with platelets or ASM and repeated clustering of ceramide and activated β1 integrin on the surface of the tumor cells using confocal fluorescence microscopy (Fig6B).

These findings indicate that the interaction between B16F10 melanoma cells and platelets results in the activation and secretion of Asm from platelets. In turn, Asm releases ceramide in tumor cells. Ceramide mediates the clustering of integrins and, thus, the adhesion of the tumor cells.

### Pre-incubation of B16F10 melanoma cells with purified ASM or C_16_ ceramide restores pulmonary tumor cell trapping in Asm-deficient mice, an effect that is revertable by inhibition of integrin function

**Figure 7:**
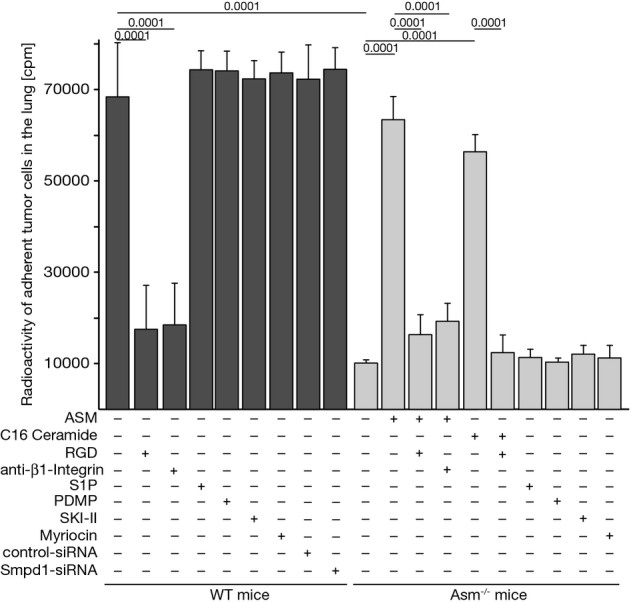
Asm-mediated β1 integrin activation mediates adhesion of tumor cells in the lung *in vivo* B16F10 tumor cells or B16F10 transfected with siRNA targeting Asm expression or with scrambled control siRNA were labeled with [^3^H]thymidine, washed, and treated (+) for 10 min with 1 U/ml ASM, 10 μM C_16_ ceramide, 10 μM S1P, 10 μM PDMP, 20 μM sphingosine kinase inhibitor SKI II, and 10 μM myriocin, or left untreated (−). The cells were then injected intravenously into wild-type or Asm-deficient mice as indicated. If indicated, 10 μg of arginine-glycine-aspartic acid (RGD) peptide or 10 μg/ml neutralizing anti-β1 integrin antibodies were added to B16F10 tumor cells together with ASM for 15 min prior injection. Mice were sacrificed 30 min after tumor cell injection, the lungs carefully flushed via the right heart, and the radioactivity in the lung as a measurement for adherent tumor cells was determined. Shown is the mean ± SD of the counts per minute (cpm) in the lung lysates from three independent experiments. Statistical significance was determined by analysis of variance (ANOVA) followed by Tukey's test for multiple comparison to determine *P*-values.

If the cascade from platelet-secreted Asm to integrin clustering and activation on tumor cells also applies to the *in vivo* events occurring in the lung, the formation of ceramide in melanoma cells should restore the metastatic potential of the tumor cells in Asm-deficient mice. To test this hypothesis, we determined tumor cell trapping in the lung 30 min after intravenous injection of the tumor cells. The results show that the tumor cells were rapidly trapped in the lungs of wild-type mice (Fig7). Tumor cell trapping was prevented by co-injection of the tumor cells with RGD peptides or neutralizing β1 integrin antibodies (Fig7). Tumor cell trapping was almost absent in the lungs of Asm-deficient mice (Fig7). Tumor cell trapping in these mice was restored by pre-incubating the tumor cells with purified ASM or C_16_ ceramide, which was again inhibited by co-incubation and co-injection with RGD peptides or antibodies neutralizing β1 integrin (Fig7). Treatment of the tumor cells with SKI II, an inhibitor of sphingosine kinases, myriocin, an inhibitor of the ceramide synthesis pathway, DL-threo-1-phenyl-2-decanoylamino-3-morpholino-1-propanol (PDMP), an inhibitor of glucosyltransferases, or pre-treatment with sphingosine 1-phosphate (S1P) prior to injection did not change trapping of the tumor cells in lungs (Fig7). In accordance, we did not find any changes of S1P upon co-incubation of B16F10 melanoma cells with wild-type platelets (Supplementary Fig S1A). Also, treatment of wild-type mice with PDMP or myriocin prior to injection of the tumor cells did not alter tumor metastasis (Supplementary Fig S1B).

Downregulation of Asm expression in B16F10 cells by 90% using siRNA technology did not affect trapping of the tumor cells in wild-type mice (Fig7) again indicating the critical role of the Asm in platelets, but not in the malignant tumor cells.

This finding confirms our hypothesis and demonstrates that the generation of ceramide on tumor cells and the subsequent clustering of integrins are sufficient to mediate tumor cell trapping and subsequent metastasis and to bypass Asm deficiency.

### Pharmacological inhibition or heterozygosity of the Asm prevents tumor metastasis

**Figure 8:**
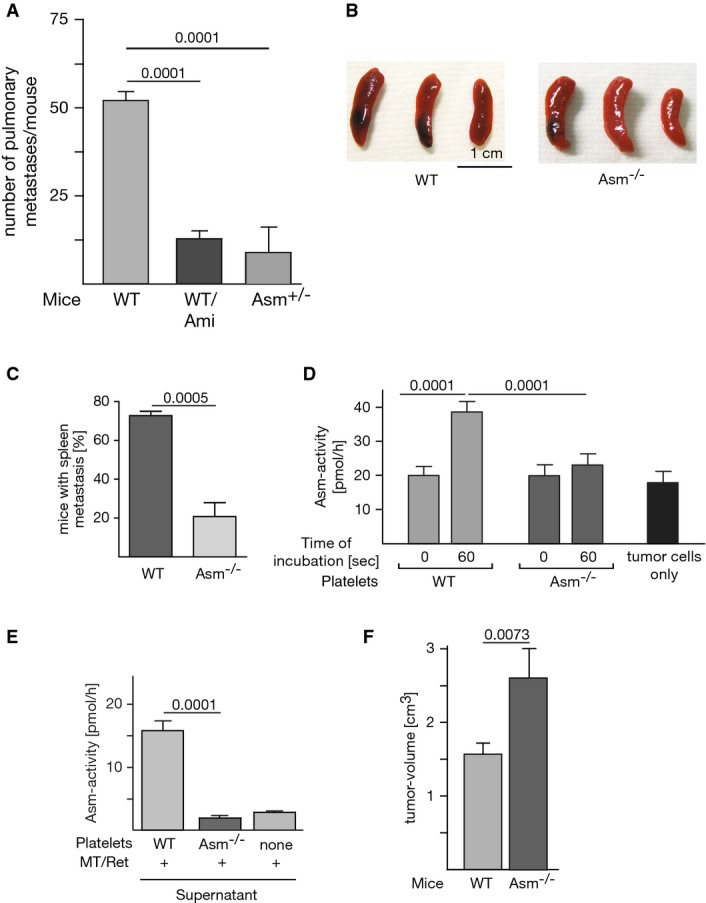
Targeting the Asm system prevents melanoma metastasis

We further tested whether a drug that inhibits acid sphingomyelinase prevents B16F10 tumor metastasis in wild-type mice *in vivo* (Fig8A). To this end, we treated C57BL/6 mice for 2 days with i.p. injections of amitriptyline. Amitriptyline is a functional blocker of the Asm, which triggers proteolytic degradation of the enzyme in lysosomes (Kolzer *et al*, 2004). Controls confirmed that the activity level of Asm in blood samples was decreased by approximately 85% upon amitriptyline treatment. The mice were intravenously injected with 1 × 10^5^ B16F10 tumor cells, and the number of lung metastases was determined after 14 days. The results show that tumor metastasis is inhibited by 75% upon treatment with the Asm inhibitor amtriptyline. To further validate the Asm as a potential target for prevention of melanoma metastasis, we employed Asm-heterozygous mice that show an approximately 50% reduction in Asm activity and do not develop Niemann-Pick disease or suffer from any other known pathology. The results show that genetic heterozygosity reduced the number of B16F10 lung metastases by 83%, very similar to the effect of amitriptyline (Fig8A).

A Amitriptyline (2 mg/kg, Ami) was intraperitoneally injected into C57BL/6 mice for five times every 12 h. Sixty minutes after the last injection, 1 × 10^5^ B16F10 tumor cells were intravenously injected. Control experiments confirmed that amitriptyline inhibited Asm activity in the blood by approximately 85%. Asm heterozygous mice were injected with 1 × 10^5^ B16F10 tumor cells. The number of lung metastases was determined after 14 days. Shown is the mean ± SD from six mice each, ANOVA followed by Tukey's test for multiple comparison. *P*-values are indicated.

B, C MT/ret cells (10^5^) suspended in 200 μl Matrigel/PBS (1:1) were injected s.c. in the left and right flanks of 8-week-old Asm-deficient mice or wild-type mice. Mice were sacrificed at day 20 after injection, and spleens were inspected for the presence of metastases. The representative photographs (B) show the results from one of four experiments, and the quantitative analysis is displayed in (C).

D Platelets were isolated from wild-type or Asm-deficient mice by density-gradient centrifugation, and 1 × 10^7^ platelets were incubated with 1 × 10^5^ MT/ret melanoma cells. The samples were lysed, and Asm activity in cell lysates was determined. In unstimulated samples (time point 0 of co-incubation), tumor cell and platelet lysates were admixed after lysis.

E To measure secretion of Asm by platelets, tumor cells and platelets were co-incubated for 30 s, the samples were pelleted, the supernatants were acidified, and the Asm activity was measured. All Asm activity measurements were performed in the presence of 100 μM Zn^2+^.

F Local tumor growth in wild-type or Asm-deficient mice at the flank was measured at day 20.

Data information: Displayed is the mean ± SD of each three or four experiments. Statistical significance was determined by *t*-test (C, E, F) or analysis of variance followed by Tukey's multiple comparisons test (D). *P*-values are indicated.

### Expression of Asm is required for spontaneous hematogenous metastasis from subcutaneous melanoma

To test whether our findings also apply to metastasis of a local, subcutaneous melanoma, we injected MT/*ret* melanoma cells subcutaneously in the left and right flanks of wild-type and Asm-deficient mice. Tumors were allowed to grow for 20 days; the mice were then sacrificed and inspected for the occurrence of metastasis. These studies revealed that hematogenous metastasis of subcutaneous melanoma to the spleen was significantly reduced in Asm-deficient mice compared to wild-type mice (Fig8B and C). To reassess whether platelet-derived Asm plays a role in this process, we tested whether Asm is activated during the interaction between MT/*ret* melanoma cells and platelets. We found that, in analogy to the previous results with B16F10 melanoma cells, co-incubation of wild-type platelets with MT/*ret* melanoma cells resulted in rapid stimulation of Asm (Fig8D). In contrast, no change in Asm activity was detected when tumor cells were incubated with Asm-deficient platelets. In addition, we determined the activity of the Asm in supernatants after co-incubation of MT/*ret* cells and wt or Asm-deficient platelets (Fig8E). The results confirm the experiments obtained with B16F10 cells and show an increased activity of the Asm in the supernatant after incubation of MT/*ret* cells with wild-type platelets, while no increase in Asm activity was detected in the supernatants from tumor cells co-incubated with Asm-deficient platelets (Fig8E). Controls show that the local growth of the MT/*ret* melanoma was even slightly faster in Asm-deficient mice (Fig8F), excluding that a growth defect of the tumor in these mice causes the lack of metastasis in this model.

## Discussion

The results of these studies indicate a novel function of Asm and ceramide: an involvement in tumor metastasis. Our experiments indicate that Asm is secreted from platelets upon their interaction with tumor cells. Secreted Asm, in turn, acts on the surface of tumor cells to catalyze the release of ceramide and to trigger the formation of ceramide-enriched membrane platforms. We have further demonstrated that these membrane platforms serve to reorganize and cluster α5β1 integrins that finally mediate adhesion. Clustering of integrins has been previously shown to be a pre-requisite for integrin activation. Although we are unable to directly investigate the activation status of α5β1 integrins in mouse cells, since no activation-specific antibody is available, we interpret the clustering as a sign of activation of α5β1 integrins. Whether ceramide only mediates integrin clustering or also modifies the conformation of integrins and thereby activates integrins is presently unknown. However, the experiments on human melanoma cells that were treated with human purified ASM or C_16_ ceramide and then subjected to immunoprecipitation with the activation-dependent HUTS-4 anti-β1 integrin antibody indicate that ceramide is sufficient to mediate β1 integrin activation.

Several lines of evidence support this model. Although B16F10 and MT/*ret* tumor cells express Asm, only the injection of these tumor cells into wild-type mice but not into Asm-deficient results in metastasis. Transplantation of wild-type platelets into Asm-deficient mice or *ex vivo* treatment of the tumor cells with purified ASM or C_16_ ceramide restored tumor metastasis. In addition, downregulation of Asm expression in tumor cells did not alter release of Asm upon co-incubation with wild-type platelets. These findings indicate that the activity of the enzyme in platelets is required for tumor metastasis and that the generation of ceramide on tumor cells is sufficient to mediate metastasis. Furthermore, we show that MT/*ret* melanoma cells also activate Asm in platelets, suggesting that the mechanism identified for hematogenous metastasis of intravenously injected B16F10 melanoma cells also applies in this model of spontaneous metastasis of a subcutaneous melanoma, underlining the relevance of our findings.

The findings of recent studies have indicated that an increase in the ceramide concentration in established tumors, in particular in lysosomes of the tumor cells, prevents tumor growth and thereby also prevents invasion and metastasis (Petersen *et al*, 2013; Paschall *et al*, 2014). These studies primarily investigated how controlling the growth of local tumors prevents tumor metastasis, whereas our studies focused on the mechanisms by which tumor cells in the blood stream attach and seed. Additional studies demonstrated that Asm plays a role in the expression of metalloprotease-1 (Bauer *et al*, 2009). However, although the expression of metalloproteases within malignant tumor cells is important for the emigration of tumor cells from the tumor mass, their migration through the tissue, and their ability to access the bloodstream, this expression does not play a role in the adhesion of tumor cells to platelets and endothelial cells, the topic of the present manuscript.

Osawa *et al* (2013) investigated the role of Asm in metastasis to the liver after the injection of colon cancer or melanoma tumor cells into the spleen. They demonstrated that tumors grow more rapidly in Asm-deficient mice because the expression of Asm is necessary for producing S1P, which triggers the accumulation of cytotoxic macrophages and promotes the production of tissue inhibitor of metalloprotease 1 (TIMP1) in the tumors, factors that reduce local tumor growth. Interestingly, Osawa *et al* did not find a reduction in tumor metastasis to the liver in Asm-deficient mice. This might be caused by the faster local growth of tumors in Asm-deficient mice. The larger size of the tumors might compensate for a reduction in hematogenous metastasis, at least in this spleen–liver metastasis model. However, it is more likely that the mechanisms mediating the attachment of tumor cells to endothelial cells of the liver differ from those mediating their attachment to endothelial cells of the lung. This hypothesis would be consistent with the clinical finding that many tumors preferentially metastasize to specific organs; this preferential metastasis suggests that the mechanisms of metastasis differ between target organs.

Our findings, demonstrating that GPIIbIIIa and CD62P are upregulated on wild-type as well as on Asm-deficient platelets upon contact with the tumor cells, indicate that the primary interaction between tumor cells and platelets is independent of Asm expression. Furthermore, we show that aggregometrical properties of platelets are not impaired by Asm deficiency. A strong activation of platelets by tumor cells was previously shown (Tohgo *et al*, 1984; Menter *et al*, 1987) to be consistent with our observation that co-incubation of B16F10 cells with platelets was sufficient to trigger degranulation. Finally, we were unable to find any platelet cluster in wild-type or Asm-deficient lungs *in vivo* after staining with anti-CD41 antibodies.

Our studies suggest a primary role of Asm-released ceramide in tumor cell metastasis, although they do not exclude the role of other sphingolipids. However, treatment of the tumor cells with inhibitors of sphingosine kinases, the ceramide synthesis pathway, DL-threo-1-phenyl-2-decanoylamino-3-morpholino-1-propanol (PDMP), glucosyltransferases, or pre-treatment with S1P are without effect on tumor cell trapping. Further, S1P concentrations did not change after co-incubation of tumor cells with platelets. These data exclude a role of glucosylceramides and S1P in the observed metastasis. In addition, we show that an Asm-dependent activation of integrins is required for tumor cell trapping. Studies from our group excluded clustering of β1 integrins upon treatment with sphingosine (not shown). However, it cannot be excluded that ceramide is converted to ceramide 1-phosphate that may, for instance via phospholipase A_2_ activation, contribute to tumor metastasis.

It seems to be very unlikely that changes associated with Niemann-Pick disease cause the observed lack of tumor metastasis, because (i) we used mice at an age (maximum 7 weeks of age) that are too young to develop signs of Niemann-Pick disease (Dhami *et al*, 2001; Lozano *et al*, 2001; Pewzner-Jung *et al*, 2014), (ii) Asm-deficient mice have normal clotting functions, normal coagulation, and normal bleeding time and platelet counts, (iii) treatment of tumor cells *ex vivo* with purified ASM or C16 ceramide was sufficient to restore tumor metastasis in Asm-deficient mice excluding an alteration of the lung as cause for the observed reduction in tumor metastasis, (iv) transplantation of wild-type platelets into Asm-deficient mice was sufficient to restore B16F10 metastasis, (v) treatment of a wild-type mice with the Asm inhibitor amitriptyline markedly reduced tumor metastasis, and (vi) Asm heterozygosity, which does not result in Niemann-Pick disease, was sufficient to reduce tumor metastasis by 83%.

Secretion of Asm from platelets has also been reported to occur after platelets have been stimulated with platelet agonists such as thrombin (Simon *et al*, 1998; Romiti *et al*, 2000). Secreted Asm may act on membranes in the vicinity of activated cells and, thus, promote adhesion. The activity of Asm on the cell surface has been demonstrated by several studies (for instance Schissel *et al*, 1998a,b; Grassmé *et al*, 2001; Rotolo *et al*, 2005; Xu *et al*, 2012). The results of these studies indicate either that secretory lysosomes containing Asm fuse with the plasma membrane and expose Asm (Grassmé *et al*, 2001; Rotolo *et al*, 2005; Herz *et al*, 2009; Xu *et al*, 2012) or that cells secrete Asm, which then binds back to the cell surface (Schissel *et al*, 1998a,b). This secretory Asm depends on Zn^2+^ for its activity, while lysosomal Asm does not depend on additional Zn^2+^. We therefore studied the Zn^2+^ dependency of the Asm that is present on tumor cells that have been co-incubated with platelets. Our findings show that the observed activity of Asm is dependent on Zn^2+^, a finding indicating that the effects of platelets on B16F10 melanoma cells are mediated by secretory Asm, which is Zn^2+^ dependent. Zn^2+^ is a nutrient and is present in the blood plasma at concentrations that allow activity of the secretory Asm (Spence *et al*, 1989). Secretory Asm has been previously shown to be active even at a neutral pH (Schissel *et al*, 1998a,b). Furthermore, pH determines the *K*_m_ but not the *V*_max_ of enzyme activity (Schissel *et al*, 1998a,b). The *K*_m_ value of sphingomyelin depends on the lipid environment and enables Asm to be active at a more neutral pH. A recent study indicated that the activity of the acid sphingomyelinase is regulated by anionic plasma membrane phospholipids (Oninla *et al*, 2014). In particular, phosphatidylglycerol and phosphatidic acid increase Asm activity, but also ceramide, diacylglycerol, and free fatty acids (Oninla *et al*, 2014). Finally, further studies by our group indicate that H^+^-ATPase clusters in ceramide-enriched membrane domains on the surface of B16F10 cells after these cells were co-incubated with platelets (Supplementary Fig S2), a finding suggesting an acidification of these domains, as previously shown by Xu *et al* (2012). Secretory Asm has been previously show to bind to cell surfaces (Dhami & Schuchman, 2004; Schissel *et al*, 1998b), allowing the enzyme to be active on the cell surface. Although the exact mechanisms of how that enzyme binds to the cell surface remain to be determined, the lipid composition of the plasma membrane, the activity of the secretory Asm at higher pH values, the presence of H^+^-ATPase in ceramide-enriched domains potentially acidifying surface microdomains, and the concentrations of Zn^2+^ in the plasma allow activity of the Asm on the cell surface.

The results of biophysical and *in vivo* studies have demonstrated that ceramide generated by acid sphingomyelinase in the outer leaflet of the cell membrane generates ceramide-enriched membrane platforms that serve the reorganization and clustering of receptor molecules (Grassmé *et al*, 2001). Previous studies have demonstrated that the activation of receptor molecules such as CD95, CD40, and DR5 results in the clustering of these receptors in ceramide-enriched membrane domains (Grassmé *et al*, 2001, 2002; Dumitru & Gulbins, 2006), whereas inactive receptors do not cluster. It has been shown that the transmembranous domain in CD40 is involved in the trapping of the receptor in ceramide-enriched domains (Bock & Gulbins, 2003); however, the molecular details mediating the specific clustering of activated receptors are unknown. Integrins also cluster upon stimulation, and it is assumed that inside-out signaling alters the conformation of integrins to initiate their trapping and clustering in distinct domains of the cell membrane (Wary *et al*, 1996; Bray *et al*, 1998; Wei *et al*, 1999). The results of the current study provide genetic and pharmacological evidence in support of the hypothesis that Asm activity triggers clustering of integrins to mediate tumor cell-adhesion.

The mechanisms that mediate the specific clustering of integrins in ceramide-enriched membrane domains in tumor cells remain to be determined. Two models may apply: First, many tumor cells exhibit a proadhesive phenotype that permits these cells to contact platelets, endothelial cells, or subendothelial structures (Gehlsen *et al*, 1992; Grossi *et al*, 1998). This proadhesive phenotype may be caused by a constitutive change of integrins into the active conformation as a consequence of malignant transformation. The adhesion of tumor cells then requires only the release of ceramide, the formation of ceramide-enriched membrane platforms, and the clustering of pre-activated integrins in these domains. In an alternative model, the activity of Asm on tumor cells may result in the activation of signaling pathways that specifically mediate the activation of integrins. Activated integrin molecules then cluster in ceramide-enriched membrane domains to mediate tumor cell adhesion. Our immunoprecipitation data on activation of β1 integrins from human melanoma cells support the latter model with an activation of integrins by ceramide.

Our findings demonstrate that Asm is required for the trapping of tumor cells in the lung. However, the results of these studies do not exclude the possibility that Asm is also involved in later events of tumor cell metastasis, such as tumor cell migration or alteration of endothelial cells to permit the exit of the tumor cells.

NK cells have been previously shown to be crucial in the antitumor activity in the blood stream, at least in mice (Wiltrout *et al*, 1985; Nieswandt *et al*, 1999). Nieswandt *et al* (1999) showed that platelets form a shield around tumor cells and thus protect them from the cytotoxic effects of NK cells. This mechanism is certainly not excluded by our data, since our studies describe molecular mechanisms of tumor cell trapping and adhesion that function independent of NK cells in the multi-step program of metastasis. Further studies from our group confirm this notion and indicate that depletion of NK cells in wild-type and Asm-deficient mice results in an increase in lung metastases in both wild-type and Asm-deficient mice, but did not change the ratio of metastasis between wild-type and Asm-deficient mice with a reduction in tumor metastasis by 77 ± 5% (*n* = 4) in Asm-deficient mice compared to the number in wild-type mice after depletion of NK cells. This indicates that the adhesion of tumor cells to lung endothelial cells depends on the Asm, while the number of tumor cells that survive after adhesion is mainly dependent on NK cells.

In the present studies, we also translated the genetic models using Asm-deficient mice to a clinical situation: We inhibited Asm by treating mice with amitriptyline. Amitriptyline interferes with the binding of Asm to membranes, and this interference results in the release of the enzyme into the lumen of lysosomes or secretory lysosomes. The enzyme is then degraded by the activity of proteases present in these vesicles (Kolzer *et al*, 2004). Our experiments demonstrate that treating mice with a dosage of amitriptyline similar to that used to treat humans is sufficient to reduce the numbers of B16F10 metastases by 75%. The concept of preventing tumor metastasis by inhibiting Asm may be particularly useful in an acute setting, such as any surgical manipulation of a tumor, but it may also be suitable for longer-term use, such as cases in which complete removal of the primary tumor is impossible to prevent further dissemination. The notion that the Asm might be a target to prevent tumor metastasis is supported by studies in Asm-heterozygous mice that show a similar reduction in Asm activity and B16F10 melanoma metastasis compared to mice treated with amitriptyline.

In summary, our findings demonstrate a novel mechanism by which the metastasis of tumor cells is regulated. The interaction of tumor cells with platelets results in the activation and release of Asm from platelets. This secreted Asm triggers the formation of ceramide-enriched membrane domains on tumor cells. These domains serve to specifically cluster activated integrin molecules on tumor cells, and these clusters finally mediate the adhesion of tumor cells to vascular structures and, thus, metastasis.

## Materials and Methods

### Mice

We used Asm (*Smpd1)*-deficient or heterozygous mice on a C57BL/6 Jackson background and their syngeneic wild-type littermates. We also performed experiments with wild-type and Asm-deficient C57BL/6 mice on Harlan background and detected an approximately 50% reduction in the absolute number of metastasized tumors compared to the Jackson background, while the genetic background did not affect the ratio of tumor metastasis between wild-type and Asm-deficient mice. All mice were bred and housed in the vivarium of the University of Duisburg-Essen, Germany. All mice were pathogen free according to the 2002 recommendations of the Federation of European Laboratory Animal Science Associations (FELASA). All procedures performed on mice were approved by the Animal Care and Use Committee of the Bezirksregierung Düsseldorf, Düsseldorf, Germany. We used age- and sex-matched siblings with an age of 6–7 weeks. At this age, we do not find obvious manifestations of Niemann-Pick disease type A. The mice serve as a model for the function of the Asm and do not suffer from Niemann-Pick disease at this age as previously shown by Lozano *et al* (2001). At this age, we did not detect macroscopical or microscopical abnormalities in the lung (Pewzner-Jung *et al*, 2014). This is consistent with previous publications from Ikegami *et al* (2003) and Dhami *et al* (2001) who showed that lung changes in Asm-deficient mice start with an age of 10 weeks.

### Mouse platelet isolation

Blood was collected by tail vein puncture and anti-coagulated with 0.38% sodium citrate, and 9 ml of phosphate-buffered saline (PBS; pH 7.2) supplemented with 3.5% bovine serum albumin (BSA), fatty acid free (Sigma-Aldrich) were added, and this mixture was incubated for 15 min at 37°C. Samples were centrifuged at 120 × *g* without brake for 20 min at room temperature, and the platelet-containing supernatant was collected. Platelets were pelleted by centrifugation with 1,340 × *g* for 10 min and were washed twice in Tyrode's buffer (134 mM NaCl, 0.34 mM Na_2_HPO_4_, 2.9 mM KCl, 12 mM NaHCO_3_, 20 mM HEPES, 5 mM glucose) supplemented with 0.35% BSA. After preparation, platelets were used immediately for the respective experiments in the indicated concentrations.

### Human platelet isolation

Human platelets were prepared from fresh blood of healthy volunteers (permission number of the Ethics Commission of the University of Duisburg-Essen, Nr. 05-2768). Of 6 ml human citrate blood was diluted in 45 ml PBS supplemented with 3.5% BSA (Fa. Roth), carefully mixed, and incubated for 20 min at 37°C. Samples were centrifuged for 20 min at 100 × *g*, the supernatant recovered, 50 nM PGE1 (Sigma) was added to prevent activation, the samples were centrifuged for 10 min at 600 × *g* at room temperature, the pellet was resuspended in 5 ml Tyrode's buffer supplemented with 50 nM PGE1, again centrifuged for 8 min at 600 × *g* at room temperature, the pellet was resuspended in 5 ml Tyrode's buffer, and platelets were counted. Finally, the cells were centrifuged for 8 min at 250 × *g* at room temperature and the platelets resuspended in Tyrode's buffer at 4 × 10^8^/ml.

### Tumor cell culture

B16F10 melanoma cells were cultured in GIBCO Minimum Essential Medium (MEM; Invitrogen, Karlsruhe, Germany) supplemented with 10 mM HEPES (pH 7.4), 2 mM L-glutamine, 1 mM sodium pyruvate, 100 μM non-essential amino acids, 100 units/ml penicillin, and 100 μg/ml streptomycin (all from Invitrogen). The cells were replaced by a freshly thawed and cultured aliquot after 4 weeks of growth so that selection of specific clones could be avoided. Cells were grown to subconfluency before all experiments. The cell line B16F10 was established in the early 1970s by Fidler (1975) by repeated retransplantation of cell clones that were isolated from lung metastases. Therefore, B16F10 is highly selected for the ability to form lung metastases. The murine melanoma cell line MT/*ret* was established from cutaneous melanoma of mice (C57BL/6) expressing the human *ret* proto-oncogene under the control of the mouse metallothionein I (MT) promoter-enhancer (Kato *et al*, 1998; Helfrich *et al*, 2010). Cells were maintained at 37°C and 5% CO_2_ in RPMI 1640 supplemented with 10% FCS, L-glutamine (2 mmol/l), and penicillin/streptomycin solution (5 units/ml; all from PAA Laboratories) and split every 3 days to ensure consistent proliferation behavior. Analyses were performed by using passages > 20. Human melanoma cells UKRV-Mel-06a (abbreviated HM cells) were grown in RPMI-1640 supplemented as above.

### Preparation of tumor cells

Cells were brought into suspension by treatment with cell dissociation solution (Becton Dickinson), washed extensively, and resuspended in HEPES/saline (H/S; 132 mM NaCl, 20 mM HEPES [pH 7.4], 5 mM KCl, 1 mM CaCl_2_, 0.7 mM MgCl_2_, and 0.8 mM MgSO_4_) in the indicated concentration.

### *In vivo* metastasis

B16F10 melanoma cells were collected as above, and the cell concentration was adjusted to 5 × 10^5^ cells/ml. Mice were intravenously injected via the tail vein with 1 × 10^5^ B16F10 (200 μl) cells. If indicated, the tumor cells were treated with 1 U/ml purified ASM (Sigma-Aldrich) or 10 μM C_16_ ceramide (Avanti Polar Lipids) for 10 min and washed once before injection. The number of tumors in the lung was determined 14 days after injection by counting macroscopically visible metastases in 1-mm-thick serial sections. To determine the trapping of the tumor cells in the lung, we labeled B16F10 tumor cells with 1 μCi/ml [^3^H]thymidine (185 GBq/mmol, Hartmann Analytic GmbH) for 48 h, washed the cells three times in H/S, intravenously injected the cells, sacrificed the mice after 30 min, and determined the radioactivity in the lung. To induce β1 integrin activation, we incubated the tumor cells for 15 min in H/S with 1 U/ml purified ASM or 10 μM C_16_ ceramide. To block integrins, we added 10 mg/ml RGD peptides or 10 μg/ml neutralizing anti-β1 integrin antibodies (clone 9EG7, BD-Pharmingen) to the cells together with the purified ASM or C_16_ ceramide. Lungs were completely digested with collagenase and pronase (Roche) for 3 hrs, and radioactivity was determined by liquid scintillation counting. In addition, tumor cells were treated for 15 min with 20 μM SKI II, an inhibitor of sphingosine kinases (Cayman), 10 μM myriocin (Sigma), an inhibitor of the ceramide synthesis pathway, or 10 μM PDMP (Sigma), an inhibitor of glucosyltransferases, or pre-treated with 5 μM sphingosine 1-phosphate (Avanti Polar Lipids) prior to injection. Further, wild-type mice were treated with PDMP (40 mg/kg i.p) (Gulbins *et al*, 2013) or myriocin (of 1.0 mg/kg i.p, once daily over 3 days) (He *et al*, 2005) prior to injection of the tumor cells.

For the transplantation experiments, wild-type or Asm-deficient platelets were isolated as above, and 5 × 10^8^ platelets were intravenously injected into wild-type or Asm-deficient mice. The mice were allowed to recover for 2 h before the injection of B16F10 melanoma cells as described above.

### Spontaneous *in vivo* metastasis

MT/*ret* cells (5 × 10^5^) suspended in 200 μl growth factor-reduced Matrigel (BD Matrigel Matrix, BD Biosciences)/PBS (1:1) were injected s.c. in the left and right flanks of 8- to 10-week-old Asm-deficient mice or wild-type controls using a 29-gauge needle syringe. Mice were sacrificed at day 20 after injection and inspected for the occurrence of metastases. Tumors and spleens were weighed and processed for morphologic analysis.

### Local tumor growth

5 × 10^5^ B16F10 or MT/*ret* tumor cells were injected subcutaneously in the flank. Tumor growth was quantified by caliper measurements every 3rd day. Mice were sacrificed at day 14 after injection, and the final tumor size was measured. B16F10 tumor cells were directly injected transcutaneously into the lung and allowed to grow for 10 days. Tumor size was determined after preparation of the lung by caliper measurement.

### Ceramide measurements

Tumor cells were collected as described above, resuspended in H/S, and warmed at 37°C for 8 min. 1 × 10^5^ tumor cells were added to 1 × 10^7^ platelets for the initiation of platelet stimulation. Controls were treated with the same volume of H/S. After the indicated time, the samples were extracted in CHCl_3_:CH_3_OH:1N HCl (100:100:1; v/v/v), and the organic phase was collected and dried. Diacylglycerol (DAG) was degraded in 0.1 N methanolic KOH at 37°C for 60 min, the samples were re-extracted, and the organic phase was dried and resuspended in 20 μl of a detergent solution containing 7.5% (w/v) n-octyl glucopyranoside and 5 mM cardiolipin in 1 mM diethylenetriaminepentaacetic acid (DETAPAC). The samples were sonicated for 10 min in a bath sonicator for the facilitation of micelle formation. Phosphorylation of ceramide was initiated by adding 70 μl of a buffer consisting of 0.1 M imidazole/HCl (pH 6.6), 0.1 M NaCl, 25 mM MgCl_2_, 2 mM ethylene glycol tetraacetic acid (EGTA), 2.8 mM dithiothreitol (DTT), 1 mM ATP, 10 μCi [^32^P]γATP, and DAG kinase (GE-Healthcare). The samples were incubated for 30 min at room temperature; the incubation was stopped by extraction in 1 ml CHCl_3_:CH_3_OH:1N HCl (100:100:1; v/v/v), 170 μl buffered saline solution (135 mM NaCl, 1.5 mM CaCl_2_, 0.5 mM MgCl_2_, 5.6 mM glucose, and 10 mM HEPES [pH 7.2]), and 30 μl of a 100 mM ethylenediaminetetraacetic acid (EDTA) solution. The lower phase was collected, dried, and dissolved in 20 μl CHCl_3_:CH_3_OH (1:1; v/v). Lipids were separated on Silica G60 thin-layer chromatography (TLC) plates with CHCl_3_:CH_3_COCH_3_:CH_3_OH:CH_3_COOH:H_2_O (10:4:3:2:1, v/v/v/v/v); plates were dried and analyzed by autoradiography. Ceramide spots were identified by co-migration with a C_16_- and C_24_- ceramide standard, scraped from the plate, and quantified by liquid scintillation counting. Comparison with a standard curve using C_16_- and C_24_-ceramide permitted the determination of ceramide amounts.

### *In situ* kinase assay of surface ceramide

Tumor cells were co-incubated with platelets as above, washed, and incubated for 15 min with 0.01 units DAG kinase in the presence of 10 μCi [^32^P]γATP and 10 μM cytochalasin B to prevent internalization of the kinase in 0.1 M imidazole/HCl (pH 6.6), 0.1 M NaCl, 25 mM MgCl_2_, and 100 μM ATP at 37°C. The samples were then extracted and analyzed as above.

### Mass spectrometry

Ceramides and S1P were extracted and quantified as recently described (Fayyaz *et al*, 2014; Pewzner-Jung *et al*, 2014). Briefly, lipid extraction of cell samples was performed using C17-ceramide and C17-S1P as internal standards. Sample analysis was carried out by rapid-resolution liquid chromatography-MS/MS using a Q-TOF 6530 mass spectrometer (Agilent Technologies, Waldbronn, Germany) operating in the positive ESI mode. The precursor ions of S1P (m/z 380.256), C17-S1P (m/z 366.240), and ceramides (C_16_-ceramide (m/z 520.508), C_17_-ceramide (m/z 534.524), C_18_-ceramide (m/z 548.540), C_18:1_-ceramide (m/z 546.524), C_20_-ceramide (m/z 576.571), C_22_-ceramide (m/z 604.602), C_24_-ceramide (m/z 632.634), C_24:1_-ceramide (m/z 630.618)) were cleaved into the fragment ions of m/z 264.270, m/z 250.252, and m/z 264.270, respectively. Quantification was performed with Mass Hunter Software (Agilent Technologies).

### Acid sphingomyelinase assays

Tumor cells and platelets were co-incubated as above. Stimulation was terminated by lysis in 250 mM sodium acetate (pH 5.0), 1.3 mM EDTA, and 1% NP-40 with or without addition of 100 μM Zn^2+^ for 5 min. The samples were diluted to 0.1% NP-40 and sonicated three times for 10 s. The samples were incubated for 30 min at 37°C with [^14^C]sphingomyelin (0.05 mCi per sample, 52 mCi/mmol; MP Biomedicals) that was resuspended after drying in 250 mM sodium acetate (pH 5.0), 1.3 mM EDTA, 0.1% NP-40. The sphingomyelinase reaction was terminated by extraction in 8 volumes of CHCl_3_:CH_3_OH (2:1, v/v), phases were separated, and radioactivity in the upper phase was determined by liquid scintillation counting. Hydrolysis of [^14^C]sphingomyelin by acid sphingomyelinase resulted in the formation of water-soluble [^14^C]choline chloride that was extracted in the upper phase and served as a measurement for the activity of the enzyme. To determine the activity of the Asm in unstimulated samples (no co-incubation of tumor cells with platelets), we lysed the tumor cells and the platelets separately, combined the lysates, and measured Asm activity.

For determination of the activity of secretory acid sphingomyelinase, platelets were co-incubated with tumor cells as above. The samples were finally centrifuged for 5 min at 1,340 × *g*, the cell pellets were discarded, the supernatants (50 μl) were added to 5 volumes of 250 mM sodium acetate (pH 5.0), 1.3 mM EDTA, and 0.1% NP-40, with or without 100 μM Zn^2+^, and the acid sphingomyelinase activity was determined as above.

### Asm surface activity by immunocomplex assays

B16F10 cells were co-incubated with wild-type or Asm-deficient platelets as above and washed in cold H/S; cell pellets were resuspended in H/S and incubated with 2 microliters of polyclonal anti-Asm antibodies (Grassmé *et al*, 2001; kindly provided by Dr. K. Sandhoff, University of Bonn, Germany) for 30 min at 4°C. Samples were washed 3 times in H/S, and pellets were then lysed in 25 mM Tris–HCl (pH 7.4), 125 mM NaCl, 10 mM EDTA, 10 mM sodium pyrophosphate, 3% Igepal, and each 10 μg/ml aprotinin and leupeptin (TN3/AL). Immunocomplexes were then immobilized by incubation with protein A/G agarose (Santa Cruz Inc.) for 30 min at 4°C. Immunocomplexes were washed three times in TN3/AL followed by three washes in 250 mM sodium acetate (pH 5.0), 1.3 mM EDTA, and 0.1% NP-40. Immunocomplexes were finally resuspended in 0.05 mCi per sample [^14^C]sphingomyelin, 250 mM sodium acetate (pH 5.0), 1.3 mM EDTA, and 0.1% NP-40 with or without addition of 100 μM Zn^2+^ and Asm activity assayed as above.

### Binding of ASM/Asm to human melanoma and B16F10

B16F10 or HM3 were collected as above, washed, resuspended in H/S, and incubated for 15 min with 1 U/ml recombinant human ASM or mouse Asm (both R&D). Cells were then washed in cold H/S, resuspended in 100 μl cold H/S, incubated for 30 min with anti-acid sphingomyelinase antibodies detecting both human and murine acid sphingomyelinase (Grassmé *et al*, 2001), washed again in H/S, and stained for 30 min with FITC-coupled anti-goat antibodies (Jackson). Cells were washed, resuspended in cold H/S, and analyzed on a Becton Dickinson FACS calibur.

### Aggregometry

For determination of platelet aggregation, light transmission was measured by using washed platelets diluted in Tyrode's buffer containing 1 mM CaCl_2_ (200 μl with 0.5 × 10^6^ platelets/μl). Platelets were incubated under stirring conditions (1,000/s) with the indicated agonist (10 μM adenine dinucleotide phosphate (ADP); 5 μg/ml collagen; 0.5 μg/ml collagen-related peptide (CRP); or 1 μM U46619) at 37°C. Transmission was recorded over a period of 10 min on a Fibrintimer 4-channel optical aggregometer (APACT Laborgeraete und Analysensysteme, Hamburg, Germany). Before measurement was begun, Tyrode's buffer was set as 100% aggregation, and washed platelet suspension was set as 0% aggregation.

### Measurement of platelet activation

B16F10 melanoma cells and platelets were prepared as described above. 1 × 10^5^ B16F10 tumor cells were incubated with 5 × 10^7^ wild-type or Asm-deficient platelets and stained with FITC-coupled anti-CD62P (Clone Wug.E9; Emfret GmbH) and PE-coupled anti-GPIIbIIIa (Clone JON/A; Emfret GmbH). Cells were washed with 500 μl PBS and analyzed by fluorescence-activated cell sorting (FACS) on a FACSCalibur flow cytometer (Becton Dickinson).

### Adhesion assays

B16F10 melanoma cells and platelets were prepared simultaneously as described above. 4 × 10^4^ B16F10 tumor cells were then incubated with 2 × 10^7^ wild-type or Asm-deficient platelets or 1 U/ml purified ASM (Sigma) for 5 min in a volume of 50 μl at 37°C. PGE1 was added at 50 ng/ml when indicated. After the addition of 500 μl MEM (Gibco) supplemented with 10 mM HEPES (pH 7.4), 2 mM L-glutamine, 1 mM sodium pyruvate, 100 μM non-essential amino acids, 100 units/ml penicillin, and 100 μg/ml streptomycin (all from Invitrogen), tumor cells were transferred to fibronectin-coated glass cover slips (Biopure) in 24-well plates (Falcon, BD Biosciences) and cultured for 60 s at 37°C. Unbound tumor cells were washed away with PBS (137 mM NaCl, 2.7 mM KCl, 10 mM sodium phosphate dibasic, and 2 mM potassium phosphate monobasic [pH 7.4]), and cells were fixed with 2% buffered PFA for 15 min. After being washed with PBS for 5 min, adherent cells were analyzed with a Leica fluorescence microscope counting the number of adherent tumor cells on the coverslip (diameter 12 mm).

### Confocal microscopy of ceramide-enriched platforms

B16F10 melanoma cells and platelets were prepared simultaneously as described above. 1 × 10^5^ B16F10 cells were incubated with 5 × 10^7^ wild-type or Asm-deficient platelets or a 45:55 mixture of wild-type and Asm-deficient platelets or with 1 U/ml purified ASM for 5 min in a volume of 50 μl. Stimulation was stopped by adding 50 μl of 4% PFA for 15 min. Cells were washed three times in H/S and incubated consecutively with 1 μg/ml anti-ceramide (Clone S58-9, Glycobiotec), 2.5 μg/ml hamster monoclonal IgG anti-α5 integrin (Clone HM alpha 5; AbD Serotec), and 10 μg/ml rat monoclonal anti-β1 integrin (Clone MB1.2; Chemicon-Millipore) for 45 min. Cells were washed twice as described above and consecutively incubated with 3 μg/ml Cy5-conjugated F(ab’)_2_ fragment donkey anti-mouse IgM, 1.5 μg/ml FITC-conjugated goat anti-Armenian hamster IgG (H + L), and 3 μg/ml Cy3-conjugated F(ab’)_2_ fragment donkey anti-rat IgG (H + L) (all from Jackson ImmunoResearch) for 45 min. After being washed twice, cells were mounted in Mowiol containing 2.5% DAPCO (Sigma) and analyzed with a Leica fluorescence microscope.

To determine *in vivo* clustering, 1 × 10^6^ B16F10 melanoma cells were i.v. injected, mice were sacrificed after 5 min, and blood was removed from the right heart and subjected to Ficoll density centrifugation. The tumor cell fraction was removed, fixed in 1% buffered PFA, and stained and analyzed as above.

Human melanoma cells and human platelets were prepared simultaneously as described above. 1 × 10^5^ human melanoma cells were incubated with 5 × 10^7^ human platelets or with 1 U/ml purified ASM for 5 min in a volume of 50 μl. Stimulation was stopped by adding 50 μl of 1% PFA for 15 min. Cells were washed three times in H/S and incubated consecutively with 1 μg/ml anti-ceramide (Clone S58-9, Glycobiotec) and 1 μg/ml mouse monoclonal IgG2b anti-human β1 integrin (active conf. HUTS-4, Millipore) for 1 h. Cells were washed twice as described above and consecutively incubated with 3 μg/ml Cy5-conjugated F(ab’)_2_ fragment donkey anti-mouse IgM (Jackson ImmunoResearch), and 3 μg/ml Alexa Flour 488 conjugated F(ab')_2_-goat anti-mouse IgG (H + L) (Molecular Probes) for 45 min. After being washed twice, cells were mounted in Mowiol containing 2.5% DAPCO (Sigma) and analyzed with a Leica fluorescence microscope.

### Treatment with amitriptyline

Mice were injected i.p. with 2 mg/kg amitriptyline 5-times, every 12 h prior to injection of the tumor cells as above.

### Analysis of active β1 integrins in human melanoma cells

Human melanoma cells UKRV-Mel-06a were grown in RPMI-1640 supplemented as above. The cells were removed from the plate by a short incubation with cell dissociation buffer, washed in PBS, and stimulated for 10 min with 1 U/ml purified ASM (Sigma) or 10 μM C_16_ ceramide. To consume or neutralize surface ceramide, respectively, we added 500 ng ceramidase (specific activity 5,000 pmol/min/μg; R&D) or 1 μg/ml anti-ceramide antibodies (Glycobiotech) during the incubations if indicated. The samples were lysed for 5 min at 4°C in TN3/AL, insoluble material was pelleted by 5-min centrifugation at 14,000 rpm at 4°C, the supernatants were subjected to immunoprecipitation with 1 μg anti-active β1 integrin antibodies (clone: HUTS-4, Millipore), immunocomplexes were immobilized for 45 min by protein A/G agarose (Santa Cruz Inc.), washed six-times in TN3/AL, eluted in 1× SDS-sample buffer, boiled for 5 min, and centrifuged, and the supernatants were separated by 7.5% SDS-PAGE, blotted onto nitrocellulose, and developed using anti-β1 integrin antibodies (EP1041Y, Abcam) and secondary AP-coupled anti-rabbit antibodies (Santa Cruz Inc.). The signals were visualized using electrochemoluminescence.

To detect active β1 integrin by flow cytometry, human melanoma cells were treated as above, washed in cold H/S, resuspended in 100 μl cold H/S, incubated for 30 min with 1 μg anti-β1 integrin antibodies clone HUTS-4, washed again in H/S, and stained for 30 min with FITC-coupled anti-mouse antibodies. Cells were washed, resuspended in cold H/S, and analyzed on a Becton Dickinson FACS calibur.

### siRNA transfections

B16F10 cells were were transiently transfected with 20 nM Alexa 488-coupled siRNA molecules against Smpd1 or controls (all from Qiagen) by electroporation at 450 V with 5 pulses, 3 ms each, using a BTX electroporator. Dead cells were removed after 24 h. This transfection resulted in reduction of Asm activity by 90%.

### Application of ceramide

C_16_ ceramide was delivered in octylgluocopyranoside (10% stock, final concentration: 0.01%). The controls were performed with the same concentration of OGP without ceramide.

### Statistical analyses

Data are presented as arithmetic means ± SD. Samples were tested for normal distribution using the David–Pearson–Stephens test. If multiple comparisons were performed, we used one-way analysis of variance (ANOVA) to test for significant differences. In this setting, for pair wise comparisons *P*-values were determined using the Tukey's test. For single comparisons, Student's *t*-tests were performed to test for significant differences. All data were obtained from independent measurements.

## Supporting Information

Supplementary Figures

Review Process File
