# Supplementary material for: Regulation of hematogenous tumor metastasis by acid sphingomyelinase
Source: EMBO Mol Med. 2015 Apr 7;7(6):714–34. doi: 10.15252/emmm.201404571 (PMC4459814; doi:10.15252/emmm.201404571)

Supplementary Information

Regulation of hematogenous tumor metastasis by acid sphingomyelinase

Alexander Carpinteiro[1,2], Katrin Anne Becker[1], Lukasz Japtok[3], Gabriele Hessler[1], Simone Keitsch[1], Miroslava Pozgajova[4], Kurt W. Schmid[5], Constantin Adams[1], Stefan Müller[6], Burkhard Kleuser[3], Michael J. Edwards[7], Heike Grassmé[1], Iris Helfrich[8], Erich Gulbins[1,7]

Departments of [1]Molecular Biology, [2]Hematology, [5]Pathology and Neuropathology, [6]Nuclear Medicine and [8]Dermatology of the University of Duisburg-Essen, Hufelandstrasse 55, 45122 Essen, Germany; [3]Institute for Nutritional Science, University of Potsdam, 14558 Nuthetal, Germany; [4]Slovak University of Agriculture, Department of Genetics and Breeding Biology, Tr. A Hlinku 2, 94976 Nitra, Slovakia; [7]Department of Surgery, University of Cincinnati, Cincinnati, USA.

Table of contents:

Page 2: Supplementary Legends

Page 3: Supplementary Figure 1: Sphingosine 1-phosphate after co-incubation of B16F10 tumor cells with platelets

Page 4: Supplementary Figure 2: H^+-ATPase clusters in ceramide-enriched membrane domains after co-incubation of tumor cells with platelets

Supplementary Figure 1: Sphingosine 1-phosphate after co-incubation of B16F10 tumor cells with platelets

Co-incubation of B16F10 melanoma cells with wild type platelets does not result in formation of S1P as determined by mass spectrometry. Likewise, *in vivo* inhibition of glucosyltransferases or sphingosine kinases by application of PDMP or SKI does not alter B16F10 tumor metastasis. Shown is the mean ± SD, from 4 independent samples, ANOVA.

Supplementary Figure 2: H^+-ATPase clusters in ceramide-enriched membrane domains after co-incubation of tumor cells with platelets

H^+-ATPase clusters in ceramide-enriched membrane domains on the surface of B16F10 cells after co-incubation of the tumor cells with platelets. Tumor cells were co-incubated with platelets for 60 seconds, fixed in 2% buffered PFA (pH 7.4) for 10 min, washed, blocked with H/S supplemented with 5% FCS and 0.01% Tween 20, washed, stained with anti-H^+-ATPase antibodies (Santa Cruz Inc.) and anti-ceramide antibodies for 45 min, washed again and stained with FITC-labelled anti-goat and Cy3-labelled anti-mouse IgM antibodies. Finally, cells were washed, embedded in Mowiol and analysed by confocal microscopy. Shown are representative studies from 3 independent experiments.

Supplementary Figure 1

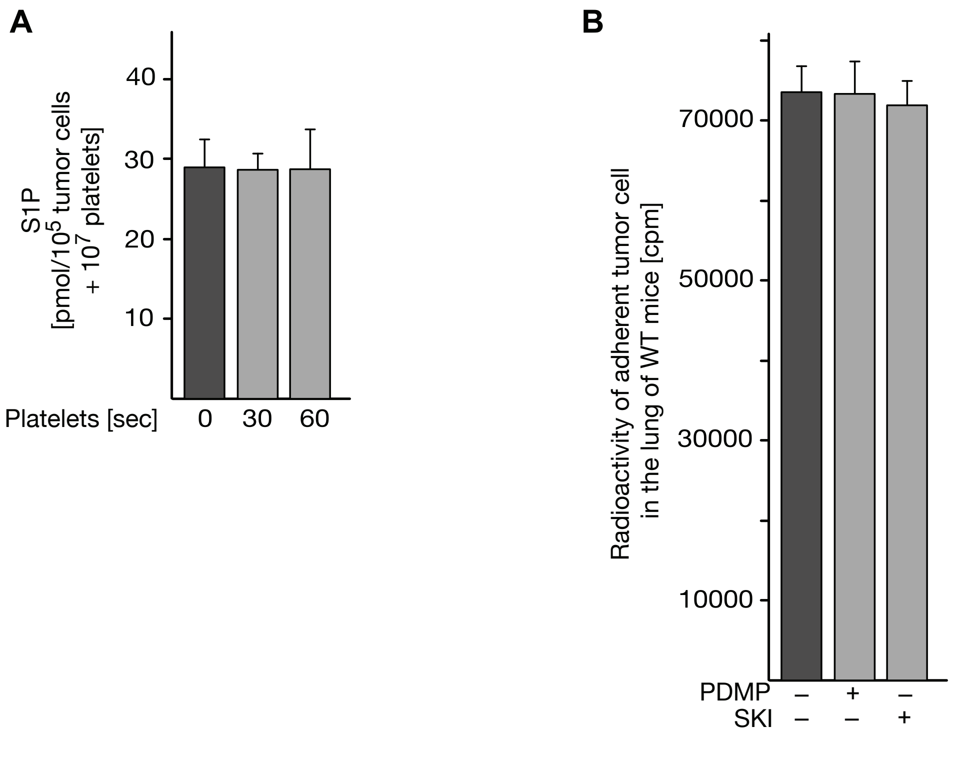

Supplementary Figure 2

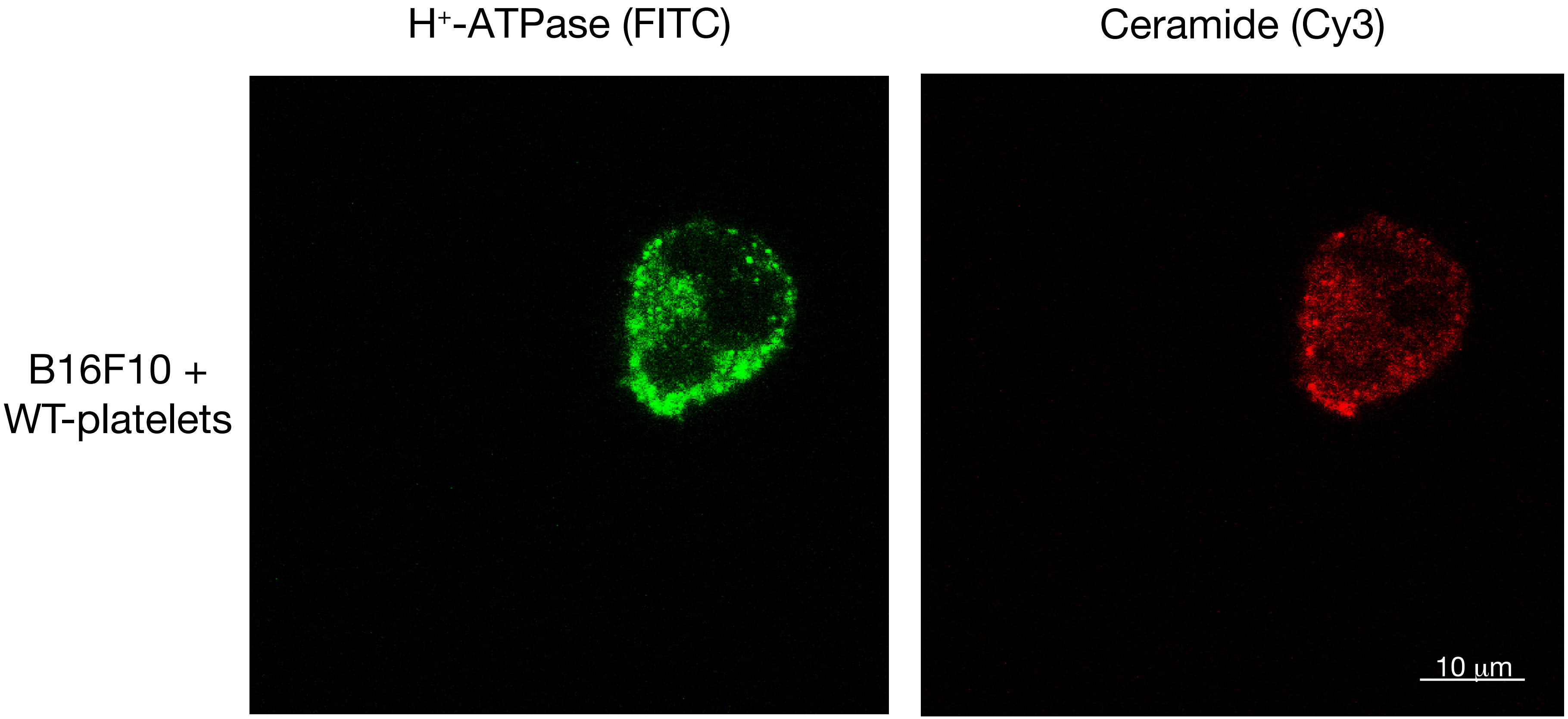

Supplement: Supplementary file 1 [file emmm0007-0714-sd1.pdf]
