# Supplementary material for: Regulation of hematogenous tumor metastasis by acid sphingomyelinase
Source: EMBO Mol Med. 2015 Apr 7;7(6):714–34. doi: 10.15252/emmm.201404571 (PMC4459814; doi:10.15252/emmm.201404571)
Supplement: Supplementary file 2 [file emmm0007-0714-sd2.pdf]

EMBO Molecular Medicine

Regulation of hematogenous tumor metastasis by acid sphingomyelinase

Alexander Carpinteiro, Katrin Anne Becker, Lukasz Japtok, Gabriele Hessler, Simone Keitsch, Miroslava Pozgajova, Kurt W. Schmid, Constantin Adams, Stefan Müller, Burkhard Kleuser, Michael J. Edwards, Heike Grassmé, Iris Helfrich, Erich Gulbins

Corresponding author: Erich Gulbins, University of Duisburg-Essen

Review timeline:

Submission date:	24 August 2014
Editorial Decision:	07 October 2014
Revision received:	02 February 2015
Editorial Decision:	17 February 2015
Revision received:	01 March 2015
Accepted:	04 March 2015

Transaction Report:

(Note: With the exception of the correction of typographical or spelling errors that could be a source of ambiguity, letters and reports are not edited. The original formatting of letters and referee reports may not be reflected in this compilation.)

Editor: Roberto Buccione

1st Editorial Decision 07 October 2014

Thank you for the submission of your manuscript to EMBO Molecular Medicine. We have now heard back from three Reviewers whom we asked to evaluate your manuscript.

We are very sorry that it has taken so long to get back to you on your manuscript. In fact, as I had previously mentioned, we experienced unusual difficulties in securing three willing and appropriate reviewers, in part due to the overlap with the vacation season.

You will see that while all three Reviewers are supportive of your work, Reviewers 1 and 4, especially, express a number of significant concerns that prevent us from considering publication at this time. I will not dwell into much detail, as the evaluations are self-explanatory and just mention a few main points. Please note that Reviewer 3 was withdrawn before s/he provided an evaluation to avoid further delays.

The main, shared concern is that additional experimentation is required to consolidate the main findings, which do not appear to be fully supported by the current data.

Reviewer 1, notes that the conclusion that Asm-derived ceramide at the plasma membrane stimulates 5/ 1 integrins is premature and also directly challenges the validity of the conclusions drawn based on the use of anti-ceramide antibodies. S/he also feels that B16 cell binding to ASM needs to be shown and, most importantly, evidence that ASM derives from platelets and ceramide from tumor cells is not definitive. This Reviewer also lists several other concerns that require your attention, among which the need to provide a convincing case for the potential translational interest

of the manuscript, with which we agree.

Reviewer 2 would like more information on the use and side effects of amitriptyline and would like you to provide more mechanistic insight on secretion of ASM from platelets. I should add that addressing Reviewer 1's requests would satisfy the latter point.

Reviewer 4 has concerns that appear complementary to those from Reviewers 1 and 2. Firstly, s/he would like to see some in vivo evidence of the formation of ceramide enriched domains with clustered integrins and would like to known whether other ASM derived ceramide metabolites involved in metastasis can be excluded. This Reviewer, similarly to Reviewer 2, would also like more mechanistic insight on secretion of ASM from platelets.

In conclusion, while publication of the paper cannot be considered at this stage, given the potential interest of your findings, we would be pleased to consider a substantially revised submission, with the understanding that the Reviewers' concerns must be addressed with additional experimental data where appropriate and that acceptance of the manuscript will entail a second round of review.

Please note that it is EMBO Molecular Medicine policy to allow a single round of revision only and that, therefore, acceptance or rejection of the manuscript will depend on the completeness of your responses included in the next, final version of the manuscript.

As you know, EMBO Molecular Medicine has a "scooping protection" policy, whereby similar findings that are published by others during review or revision are not a criterion for rejection. I understand that in this case, to address the above might entail a significant amount of additional work and time and might be technically challenging. However, I do ask you to get in touch with us after three months if you have not completed your revision, to update us on the status. Please also contact us as soon as possible if similar work is published elsewhere.

I look forward to seeing a revised form of your manuscript as soon as possible.

***** Reviewer's comments *****

Referee #1 (Remarks):

In this manuscript, the authors demonstrate a role for platelet derived acid sphingomyelinase (Asm) in tumor adhesion and hematogenous dissemination of melanoma cells in mice. The researchers use a combination of genetic and pharmacologic approaches to implicate Asm in the metastatic seeding of melanoma cells and retention in lung or spleen. The authors define a role for platelet derived Asm on ceramide raft formation and fibrinogen receptor activation in the melanoma cells. Further studies suggest a role of ceramide in integrin signaling in tumor cell adhesion. There are important achievements in this manuscript; however, there are several concerns that are presented below.

Major concerns:

1)The authors show that platelets from Asm-/- animals have normal activation and degranulation kinetics, resulting in the release of Asm. However, all of the Asm activity assays are carried out in acidic conditions. In their model, the enzyme must have activity at neutral pH in order to generate ceramide at the plasma membrane. This conclusion cannot be made from the data presented in the paper.
2)Related to point #1, the authors measure ceramide in the supernatant but claim that ceramide at the plasma membrane is responsible for integrin activation. The authors do not show any ceramide measurements from cells. As it stands, the conclusion that Asm derived ceramide at the plasma membrane stimulates 5/ 1 integrins is not well supported and premature. Along these lines, Fig 6A on integrin clustering: please show a field with several cells/field.
3)The authors rely solely on anti-ceramide antibodies and qualitative confocal imaging to support one of the most important conclusions of the paper. The ability of this antibody to detect other lipids renders these conclusions tentative at best.
4)Also, the authors need to show that B16 cells bind ASM if the model is correct.
5)The authors do not investigate the role of platelet aggregation in formation of metastatic emboli in

their model. Therefore, the conclusion that melanoma cell do not form lung mets, may primarily be a result of the defective platelets and not from an adherence defect in the tumor cells. Asm-/- mice have significant interstitial lung disease, which may significantly impact tumor cell seeding in the lung tissue.
6)The authors rely almost exclusively on the homozygous Asm mice. Niemann-Pick cells have serious endocytosis defects. It would have been much more convincing if the authors show results in heterozygous mice.
7)The authors provide a thorough description of the role of Asm in mice, but do not attempt to extend their findings into a relevant model of human disease. Does treatment of human melanoma cells with Asm result in integrin activation? Are there any clinical correlations (e.g. outcome of cancers in heterozygous Niemann-Pick carriers)? Along the same lines, NPD-B patients have thrombocytopenia.
8)Fig 3: Fig 3A how do you know ASM derives from platelets and not tumor cells? Fig 3B: how do you know that ceramide comes from the tumor cells? What is the difference between 3A and 3C?

Other points and concerns:
1)There is significant literature on possible roles of acid sphingomyelinase and ceramide in metastasis. Osawa et. al. demonstrated a strong mechanistic role for Asm in the regulation of TIMP1 activity and metastasis of colon cancer cells. Additionally, recent work by Petersen et. al. has demonstrated that Asm regulation of lysosomal homeostasis is an attractive target for therapeutic intervention in invasive cancers. Paschelli et al studied ceramide in metastasis of colon and breast cancer cells. These papers are not cited or discussed in the current manuscript [1,2,3]. Please include some discussion of how the current work adds to the field.
2)There are no scale bars in Figure 1A, Figure 6A, Figure 6B, Figure 6D, or Figure 8A.
3)Fig 3B y axis: are the levels of ceramide corrected for protein or Lipid?
4)On page 16, the authors mention a role for NK cells in survival of mets and reference data as not shown. This data could add to the paper significantly.
5)p10: tumor cell 'arrest' in the lung is not clear terminology; perhaps trapping.
The blot in Figure 6E is not exposed enough. Please show a longer exposure of this blot.P11; why is melanoma metastasizing to spleen a more 'physiologic model'?
6)P7: what is the evidence that ASM is found in secretory lysosomes?

1.Petersen, N.H., et al., Transformation-associated changes in sphingolipid metabolism sensitize cells to lysosomal cell death induced by inhibitors of acid sphingomyelinase. Cancer Cell, 2013. 24(3): p. 379-93.
2.Osawa, Y., et al., Liver acid sphingomyelinase inhibits growth of metastatic colon cancer. J Clin Invest, 2013. 123(2): p. 834-43.
3. Paschelli et al BMC Cancer 2014, 14:24

Referee #2 (Remarks):

This is an outstanding study showing a role for ASM in tumor metastasis and specifically, a role for ASM secreted from platelet cells. The study is performed at an excellent level of technical competence, and having read it twice, I cannot find any obvious flaws! I only have two minor comments that should be addressed.

The authors suggest that amitriptyline could be used as a therapy. However, this needs more discussion. The authors state that amitriptyline is used at a concentration similar to that used in humans, but do not reference this and nor do they state what disease amitriptyline is used in. Are there off-target effects of amitriptyline? If so, is this a feasible treatment, or do more selective ASM inhibitors need to be developed? Thus, the potential use of amitriptyline needs to be discussed in more detail.

Also, the authors should expand a little on the mechanism of secretion of ASM from platelets. Would inhibiting ASM secretion be another possible therapeutic approach?

Referee #4 (Comments on Novelty/Model System):

The manuscript describes a novel interaction between host platelets and melanoma cancer cells. Specifically a model is proposed in which platelets release ASM, which promotes formation of ceramide-enriched platforms in the melanoma cells, into which integrins are clustered. This is required for hematogenous metastasis related to enhanced invasive properties of the melanoma cells. There are a number of very well controlled experiments to demonstrate this mechanistic model along with two animal models of metastasis in mice. The evidence for the role of ASM in this process is interesting and provocative and should have significant medical impact if correct e.g. in targeting ASM therapeutically with chemical inhibitors.

However, there are a number of experiments that are required to establish a higher level of proof for the proposed model (see below). Most of the experiments are performed on isolated platelets and melanoma cells in vitro and the observation require direct observation in vivo. In addition, mechanistic evidence for how melanoma cells cause release of ASM from platelets is lacking. Finally, further metabolism of ceramide and exclusion of metabolised products from inducing metastasis are required.

The recommendation is too pursue revision of the manuscript with a favourable view.

Referee #4 (Remarks):

The manuscript describes a novel interaction between host platelets and melanoma cancer cells. Specifically a model is proposed in which platelets release ASM, which promotes formation of ceramide-enriched platforms in the melanoma cells, into which integrins are clustered. This is required for hematogenous metastasis related to enhanced invasive properties of the melanoma cells. There are a number of very well controlled experiments to demonstrate this mechanistic model along with two animal models of metastasis in mice. The evidence for the role of ASM in this process is interesting and provocative and should have significant medical impact if correct e.g. in targeting ASM therapeutically with chemical inhibitors.

However, there are a number of experiments that are required to establish a higher level of proof for the proposed model (see below). Most of the experiments are performed on isolated platelets and melanoma cells in vitro and the observation require direct observation in vivo. In addition, mechanistic evidence for how melanoma cells cause release of ASM from platelets is lacking. Finally, further metabolism of ceramide and exclusion of metabolised products from inducing metastasis are required.

Points

i. Demonstrate formation of ceramide enriched domains with clustered integrins in B16F10 and B16F10 in vivo and absence in cells injected into ASM-/- mice; by retrieving cells from mice.
ii. Provide some details as to the mechanism by which B16F10 and B16F10 cause the release of ASM from platelets
iii. Exclude other ASM derived ceramide metabolites involved in metastasis e.g. using ceramide synthase inhibitors, SphK inhibitors and utilise relevant knockouts to establish whether dissemination is affected and whether this is specific to cancer cells or fibroblasts or platelets. For instance, there is evidence from Albinet et al and Ponnusamy et al. for a role for S1P in metstastasis and S1P can obviously be derived from ceramide.

1st Revision - authors' response 02 February 2015

Referee #1 (Remarks):

In this manuscript, the authors demonstrate a role for platelet derived acid sphingomyelinase (Asm) in tumor adhesion and hematogenous dissemination of melanoma cells in mice. The researchers use a combination of genetic and pharmacologic approaches to implicate Asm in the metastatic seeding of melanoma cells and retention in lung or spleen. The authors define a role for platelet derived Asm on ceramide raft formation and fibrinogen receptor activation in the melanoma cells. Further studies suggest a role of ceramide in integrin signaling in tumor cell adhesion. There are important achievements in this manuscript; however, there are several concerns that are presented below.

Major concerns:

1) The authors show that platelets from Asm-/- animals have normal activation and degranulation kinetics, resulting in the release of Asm. However, all of the Asm activity assays are carried out in acidic conditions. In their model, the enzyme must have activity at neutral pH in order to generate ceramide at the plasma membrane. This conclusion cannot be made from the data presented in the paper.

Response:

Studies by I. Tabas et al. have previously demonstrated that secretory acid sphingomyelinase is active even at a neutral pH (Schissel SL, Jiang X, Tweedie-Hardman J, Jeong T, Camejo EH, Najib J, Rapp JH, Williams KJ, Tabas I (1998) Secretory sphingomyelinase, a product of the acid sphingomyelinase gene, can hydrolyze atherogenic lipoproteins at neutral pH. Implications for atherosclerotic lesion development. J Biol Chem 273: 2738-2746). To be active, secretory Asm requires Zn^{2+}. In studies determining whether the activity of Asm after the co-incubation of tumor cells and platelets is dependent on Zn^{2+}, we have now demonstrated that it is secreted Asm that binds to the surface of the B16F10 tumor cells. We obtained this finding by measuring the Zn^{2+} dependency of the Asm found in the supernatant of co-incubated tumor cells and platelets and of the Asm that is immunoprecipitated from the cell surface after the co-incubation of tumor cells and platelets. The results of these studies clearly show the dependency of Asm activity on Zn^{2+}, demonstrate the role of secretory Asm, and explain the activity of Asm on the cell surface.

Furthermore, Callahan et al. (Callahan JW, Jones CS, Davidson DJ, and Shankaran P (1983) The active site of lysosomal sphingomyelinase: evidence for the involvement of hydrophobic and ionic groups. J Neurosci Res 10: 151-163) have previously demonstrated that pH determines the K_m of lysosomal Asm but not the maximal velocity (V_{max}) for sphingomyelin hydrolysis. Because lipid modification on the cell surface (for instance, the oxidation of lipids) greatly increases the amount of sphingomyelin that is accessible to lysosomal Asm, it must be assumed that even the lysosomal Asm that is present on the cell surface after secretory lysosomes have fused with the plasma membrane is active at more neutral pH.

Although these studies have clearly shown that Asm is active on the cell surface within a broad pH spectrum, studies by Pin Lan Li's group found that the exposure of Asm on the cell surface results in formation of membrane domains that contain active proton pumps, which mediates an acidification of the small surface domain containing Asm (Xu M, Xia M, Li X-X, Han W-Q, Boini KM, Zhang F, Zhang Y, Ritter JK, Li P-L (2012) Requirement of translocated lysosomal V1 H(+)-ATPase for activation of membrane acid sphingomyelinase and raft clustering in coronary endothelial cells. Mol Biol Cell 23: 1546-1557). We therefore determined whether a co-localization of ceramide and H^+-

ATPase on the surface of B16F10 cells occurs after co-incubation of tumor cells and platelets as a marker of acidification. The results of these confocal microscopy studies show that ceramide and H^+-ATPase cluster and co-localize in stimulated cells. We also attempted to directly measure the pH on the cell surface by using a gel containing the fluorescent dye OG488, as described by P.L. Li, but these experiments failed because it is impossible to obtain a proper contact between tumor cells and platelets in a gel. Furthermore, the gel itself activated the platelets, thus excluding a direct measurement of the pH.

To address these issues, we changed the Discussion section of our manuscript as follows:

The activity of Asm on the cell surface has been demonstrated by several studies (for instance Schissel et al, 1998a,b; Grassmé et al, 2001, Rotolo et al, 2005; Xu et al, 2012). The results of these studies indicate either that secretory lysosomes containing Asm fuse with the plasma membrane and expose Asm (Grassmé et al, 2001, Rotolo et al, 2005; Herz et al, 2009; Xu et al, 2012) or that cells secrete Asm, which then binds back to the cell surface (Schissel et al, 1998a,b). This secretory Asm depends on Zn^{2+} for its activity, while lysosomal Asm does not depend on additional Zn^{2+}. We therefore studied the Zn^{2+} dependency of the Asm that is present on tumor cells that have been co-incubated with platelets. Our findings show that the observed activity of Asm is dependent on Zn^{2+}, a finding indicating that the effects of platelets on B16F10 melanoma cells are mediated by secretory Asm, which is Zn^{2+} dependent. Secretory Asm has been previously shown to be active even at a neutral pH (Schissel et al, 1998a,b). Furthermore, pH determines the K_m but not the V_{max} of enzyme activity (Schissel et al, 1998a,b). The K_m value of sphingomyelin depends on the lipid environment and enables Asm to be active at a more neutral pH. Finally, further studies by our group indicate that H^+-ATPase clusters in ceramide-enriched membrane domains on the surface of B16F10 cells after these cells were co-incubated with platelets (Supplementary Figure 2), a finding suggesting an acidification of these domains, as previously shown by Xu et al. (2012).

2) Related to point #1, the authors measure ceramide in the supernatant but claim that ceramide at the plasma membrane is responsible for integrin activation. The authors do not show any ceramide measurements from cells. As it stands, the conclusion that Asm derived ceramide at the plasma membrane stimulates β1-integrin is not well supported and premature. Along these lines, Fig 6A on integrin clustering: please show a field with several cells/field.

Response:

We apologize for not making this conclusion more clearly in the original manuscript. We always measured ceramide levels in cell extracts, never in the supernatant, because ceramide is not water-soluble. This is now clearly indicated on the legend. We have rewritten the Methods section.

We measured Asm in the supernatant and in the cellular fraction. As described above, Asm is secreted by activated platelets before it binds to the surface of tumor cells, and during that time it is detectable in the supernatant. We now provide direct evidence of the secretion of Asm by measuring the activity of Asm in the tumor cells stimulated with platelets in the presence or absence of Zn^{2+}. The activity of secreted Asm requires the addition of Zn^{2+}.

We have now used mass spectrometry to further measure ceramide levels in tumor cells themselves or in tumor cells co-incubated with platelets. The results obtained by mass spectrometry are the same as those obtained by the kinase assays, i.e., an increase in ceramide concentrations after the tumor cells have been co-incubated with wild type platelets, whereas incubation with Asm-deficient platelets did not alter ceramide levels. These findings are shown in Fig. 3B of the revised manuscript.

Furthermore, we determined ceramide levels exclusively on the cell surface by using a ceramide kinase assay on intact cells. The results of this assay confirmed a marked increase in ceramide levels on the cell surface after the tumor cells were co-incubated with platelets. These findings are presented in Fig. 3D.

Similar data were obtained for human melanoma cells: Incubation of these cells with human platelets resulted in the formation of ceramide, the release of Zn^{2+}-dependent Asm into the supernatant and Zn^{2+}-dependent activity of Asm on cell surfaces (Fig. 3E).

We also performed very detailed studies on integrins with human melanoma cells. The fact that the β1 integrin antibody clone HUTS-4 (Millipore) binds only to the active form of β1 integrin allows us to determine the activation of β1 integrin by using fluorescence-activated cell sorting (FACS) analysis or Western blot analysis after immunoprecipitation of the active β1 integrin. The results of these studies demonstrate that incubation of human melanoma cells with C_{16} ceramide or with sphingomyelinase is sufficient to induce the activation of β1 integrin. Co-incubation with either a ceramidase or an anti-ceramide antibody prevented the effect of treatment with C_{16} ceramide or sphingomyelinase on the activation of β1 integrin. This finding demonstrates that the generation of ceramide is sufficient to activate β1 integrin. These data are now provided in Fig. 6F and G.

Finally, addition of human or mouse recombinant Asm to human melanoma or B16F10 cells, respectively, resulted in binding of the Asm to the tumor cell surfaces (Fig. 3F).

Fig. 6A was obtained by confocal microscopy. Because the cluster does not localize to a particular pole of the cell, it is technically impossible to provide a picture with sufficient magnification to correctly show all cells with clusters, because many clusters are out of focus. We therefore quantified the percentage of cells positive for ceramide/β1 integrin clusters in 3 independent experiments. These results are presented in Fig. 6C.

3) The authors rely solely on anti-ceramide antibodies and qualitative confocal imaging to support one of the most important conclusions of the paper. The ability of this antibody to detect other lipids renders these conclusions tentative at best.

Response:

We have now used mass spectrometry and kinase assays to measure ceramide levels. These results are presented in Fig. 3B.

Furthermore, we determined ceramide levels exclusively on the cell surface by using an in situ ceramide kinase assay on intact cells. The results of these assays confirm a marked increase in ceramide levels on the surface of tumor cells that have been co-incubated with platelets. These findings have been added to Fig. 3 (Fig 3D).

The use of human cells allowed us to directly show the activation of β1 integrins by ceramide without using anti-ceramide antibodies. Western blotting was used to quantify the activation of β 1 integrin. These studies demonstrated that generating ceramide by co-incubating tumor cells with sphingomyelinase or by applying C_{16} ceramide is sufficient to activate β1 integrins. Co-incubation of the cells either with ceramidase to reduce surface ceramide levels or with anti-ceramide antibodies to neutralize ceramide on the cell surface prevented the activation of β1 integrin. These findings are now shown in Fig. 6F.

To measure exclusively the cell surface activity of Asm after co-incubating tumor cells with wild type platelets or with Asm-deficient platelets, we washed the samples in cold buffer after the indicated incubation to remove Asm from the supernatant and then incubated the cells with anti-Asm antibodies for 30 min at 4°C, washed and lysed the cells. We then immunoprecipitated the Asm and measured its activity with an immuno-complex assay using [^{14}C]sphingomyelin as substrate. The results confirm the localization of Asm on the surface of tumor cells that have been co-incubated with wild type platelets, whereas co-incubation of tumor cells with Asm-deficient platelets did not result in the exposure of any measurable Asm activity on the cell surface (Fig. 3C).

4) Also, the authors need to show that B16 cells bind ASM if the model is correct.

Response:

We have now measured the binding of recombinant human or murine Asm to HM3 and B16F10 cells. Using flow cytometry studies, we were able to quantify binding. The results of these studies

demonstrate the binding of Asm onto the cell surface and are presented in Fig. 3F.

5) The authors do not investigate the role of platelet aggregation in formation of metastatic emboli in their model. Therefore, the conclusion that melanoma cell do not form lung mets, may primarily be a result of the defective platelets and not from an adherence defect in the tumor cells. Asm-/- mice have significant interstitial lung disease, which may significantly impact tumor cell seeding in the lung tissue.

Response:

We performed detailed studies of platelet functions (Fig. 4). The results of these studies showed that Asm-deficient platelets exhibit normal clotting functions. The mice also exhibit normal coagulation time, normal bleeding time, and normal platelet counts.

Most importantly, we have previously shown that treating tumor cells *ex vivo* with either Asm or C_{16} ceramide is sufficient to restore tumor metastasis in Asm-deficient mice, which is prevented by treatment with RGD petides.

Likewise, transplantation of Asm-deficient mice with wild type platelets is sufficient to restore metastasis of B16F10 melanoma cells in Asm-deficient mice.

These findings exclude an alteration of the lung as the cause of the observed reduction in tumor metastasis. In addition, we have now investigated serial lung sections from tumor-injected wild type and Asm-deficient mice to determine whether platelet clumps exist. For easy identification, the tumor cells were labeled with CFSE and the platelets were labeled with anti-CD41. However, we did not find any platelet aggregates, a finding suggesting that tumor metastasis is not mediated by platelet clustering. This finding is now mentioned in section Discussion.

The notion that Asm plays a crucial role in tumor metastasis is also supported by the results of experiments using amitriptyline to inhibit Asm activity in wild type mice. Amitriptyline reduced Asm activity in wild type mice by 85% and inhibited tumor metastasis by 75%. The findings of these studies have now been confirmed by the results of experiments using Asm-heterozygous mice. These mice exhibit a reduction of approximately 50% in Asm activity, an effect very similar to that of amitriptyline. Asm-heterozygous mice exhibit no alterations in the lung or changes in platelet functions. Metastasis was reduced by 83% in Asm-heterozygous mice, a finding confirming the crucial role of Asm in tumor metastasis.

6) The authors rely almost exclusively on the homozygous Asm mice. Niemann-Pick cells have serious endocytosis defects. It would have been much more convincing if the authors show results in heterozygous mice.

Response:

We agree and we have now performed these studies. The results show a reduction of 83% in tumor metastasis in Asm-heterozygous mice (and of 75% in amitriptyline-treated mice).

7) The authors provide a thorough description of the role of Asm in mice, but do not attempt to extend their findings into a relevant model of human disease. Does treatment of human melanoma cells with Asm result in integrin activation? Are there any clinical correlations (e.g. outcome of cancers in heterozygous Niemann-Pick carriers)? Along the same lines, NPD-B patients have thrombocytopenia.

Response:

We have now performed detailed studies with human melanoma cells.

As outlined above, the results of these studies indicate that treating human melanoma cells with sphingomyelinase or with C_{16} ceramide activates β1 integrin, an effect prevented by co-incubating tumor cells with ceramidase or with anti-ceramide antibodies. These results are now presented in Fig. 6F.

Furthermore, we incubated human melanoma cells with platelets and repeated the clustering of ceramide and activated β1 integrin on the surface of the tumor cells. Representative results are shown in Fig. 6G of the revised manuscript.

To the best of our knowledge, no clinical data exist about the incidence of melanoma metastasis in Niemann-Pick patients or heterozygous humans. Although this information would be certainly interesting, performing a retrospective study with exact longitudinal data, time considerations, malignancy grade of the melanoma, etc., would be extremely difficult given the small proportion of identified Niemann-Pick heterozygous individuals who also have melanoma.

8) Fig 3: Fig 3A how do you know ASM derives from platelets and not tumor cells? Fig 3B: how do you know that ceramide comes from the tumor cells? What is the difference between 3A and 3C?

Response:

We have now used small interfering RNA (siRNA) technology to suppress Asm expression in B16F10 tumor cells. Suppression of 90% was confirmed by measurements of enzymatic activity. This siRNA-mediated suppression did not alter the release of Asm after co-incubation of tumor cells with wild type platelets, nor did it affect the activity of surface Asm as determined by immuno-complex enzyme assays (Fig. 3G). Suppression of the Asm also did not affect metastasis of the tumor cells in wild type mice *in vivo* (Fig. 6H).

In contrast, co-incubation of Asm-containing B16F10 cells with Asm-deficient platelets failed to release Asm in the supernatant. Likewise, no Asm was present on the surface of the cells, as determined by measurements of surface Asm activity (Fig. 3C).

These studies clearly demonstrate that Asm is derived from platelets.

Fig. 3A shows the total Asm activity in the samples, including the supernatants and cells. Fig. 3C shows the activity of Asm in the supernatant after removal of the cells by centrifugation.

Other points and concerns:

1)There is significant literature on possible roles of acid sphingomyelinase and ceramide in metastasis. Osawa et. al. demonstrated a strong mechanistic role for Asm in the regulation of TIMP1 activity and metastasis of colon cancer cells. Additionally, recent work by Petersen et. al. has demonstrated that Asm regulation of lysosomal homeostasis is an attractive target for therapeutic intervention in invasive cancers. Paschelli et al studied ceramide in metastasis of colon and breast cancer cells. These papers are not cited or discussed in the current manuscript [1,2,3]. Please include some discussion of how the current work adds to the field.

Response:

We have now discussed these papers. We did not previously discuss them because they do not directly relate to platelet-mediated adhesion of tumor cells either in the blood or to endothelial cells.

Osawa et al. investigated the role of Asm in metastasis to the liver after injection of colon cancer cells or melanoma cells (mentioned in the discussion, no data are shown) into the spleen of mice. They demonstrated that tumors grow more rapidly in Asm-deficient mice because the expression of Asm is necessary for producing sphingosine 1-phosphate (S1P), which triggers the accumulation of cytotoxic macrophages and promotes the production of tissue inhibitor of metalloprotease 1 (TIMP1) in the tumors, factors that reduce local tumor growth. Interestingly, Osawa et al. did not find a reduction of tumor metastasis to the liver in Asm-deficient mice, perhaps because local tumors are larger in Asm-deficient mice and thus compensate for a reduction in hematogenous

metastasis. However, it is more likely that the mechanisms mediating the attachment of tumor cells to endothelial cells of the liver differ from those mediating their attachment to endothelial cells of the lung. This hypothesis would be consistent with the clinical finding that many tumors preferentially metastasize to specific organs; this preferential metastasis suggests that the mechanisms of metastasis differ between target organs.

It is possible that TIMP1 also contributes to the emigration of tumor cells after adhesion in the lung, although this possibility seems unlikely because using C_{16} ceramide to activate integrins on tumor cells *in vitro* and then injecting them into Asm-deficient mice is sufficient to fully restore metastasis in these mice.

Bock et al. demonstrated that the expression of metalloprotease-1 by Asm regulates interleukin (IL)-1 stimulation. These very interesting studies apply to the emigration of tumor cells from the tumor mass, the migration of tumor cells through the tissue, and the ability of tumor cells to access the blood stream. However, these proteases do not play a role in the adhesion of tumor cells to endothelial cells, which is the focus of the present manuscript.

The findings of recent studies suggest that the lysosome is the target for treatment of invasive tumors (Petersen et al, 2013). However, these studies investigated local invasion, whereas our studies determined the role of the Asm-ceramide system in hematogenous metastasis.

Paschall et al. demonstrated that a ceramide analog kills both primary tumor and metastasized colon cancer and breast cancer tumor cells. The authors concluded the following: “Although LCL85 possesses direct anti-tumor cytotoxicity (Figure 1) that might contribute to the observed tumor suppression, it is possible that LCL85 might also sensitize the tumor cells to apoptosis induction by FasL of host immune cells, particularly CD8+ CTLs. ... Therefore, LCL85 might sensitize colon carcinoma cells to host FasL+ CTL-mediated tumor suppression.” In addition, these authors reported that the drug LCL85 suppresses the growth and weight of primary mammary tumors before metastasis. Treating mice with LCL85 after removal of the primary tumor also reduces tumor metastasis. This effect is probably due to the marked effect of LCL85 on growth of local remainings of the tumor preventing their metastasis. The results of these studies demonstrate the effect of ceramide on the growth of tumor cells but do not elucidate the effect of platelet Asm on tumor cells during hematogenous metastasis.

These manuscripts are now discussed in our manuscript as follows:

The findings of recent studies have indicated that an increase in the ceramide concentration in established tumors, in particular in lysosomes of the tumor cells, prevents tumor growth and thereby also prevents invasion and metastasis (Petersen et al, 2013; Paschall et al, 2014). These studies primarily investigated how controlling the growth of local tumors prevents tumor metastasis, whereas our studies focused on the mechanisms by which tumor cells in the blood stream attach and seed. Additional studies demonstrated that Asm plays a role in the expression of metalloprotease-1 (Bauer et al, 2009). However, although the expression of metalloproteases within malignant tumor cells is important for the emigration of tumor cells from the tumor mass, their migration through the tissue, and their ability to access the bloodstream, this expression does not play a role in the adhesion of tumor cells to endothelial cells, the topic of the present manuscript.

Osawa et al. (2013) investigated the role of Asm in metastasis to the liver after the injection of colon cancer and melanoma cells into the spleen. They demonstrated that tumors grow more rapidly in Asm-deficient mice because the expression of Asm is necessary for producing S1P, which triggers the accumulation of cytotoxic macrophages and promotes the production of tissue inhibitor of metalloprotease 1 (TIMP1) in the tumors, factors that reduce local tumor growth. Interestingly, Osawa et al. did not find a reduction of tumor metastasis to the liver in Asm-deficient mice. This might be caused by the faster local growth of tumors in Asm-deficient mice. The larger size of the tumors might compensate for a reduction in hematogenous metastasis. However, it is more likely that the mechanisms mediating the attachment of tumor cells to endothelial cells of the liver differ from those mediating their attachment to endothelial cells of the lung. This hypothesis would be consistent with the clinical finding that many tumors preferentially metastasize to specific organs; this preferential metastasis suggests that the mechanisms of metastasis differ between target organs.

The following references have now been cited:

1. Petersen NH, Olsen OD, Groth-Pedersen L, Ellegaard AM, Bilgin M, Redmer S, Ostenfeld MS, Ulanet D, Dovmark TH, Lønborg A, Vindeløv SD, Hanahan D, Arenz C, Ejsing CS, Kirkegaard T, Rohde M, Nylandsted J, Jäättelä M (2013) Transformation-associated changes in sphingolipid metabolism sensitize cells to lysosomal cell death induced by inhibitors of acid sphingomyelinase Cancer Cell 24: 379-393.

2. Osawa Y, Suetsugu A, Matsushima-Nishiwaki R, Yasuda I, Saibara T, Moriwaki H, Seishima M, Kozawa O (2013) Liver acid sphingomyelinase inhibits growth of metastatic colon cancer. J Clin Invest 123: 834-843.

3. Bauer J, Huy C, Brenmoehl J, Obermeier F, Bock J (2009) Matrix metalloproteinase-1 expression induced by IL-1beta requires acid sphingomyelinase. FEBS Lett 583: 915-920.

4. Paschall AV, Zimmerman MA, Torres CM, Yang D, Chen MR, Li X, Bieberich E, Bai A, Bielawski J, Bielawska A, Liu K (2014) Ceramide targets xIAP and cIAP1 to sensitize metastatic colon and breast cancer cells to apoptosis induction to suppress tumor progression. BMC Cancer 14:24. doi: 10.1186/1471-2407-14-24.

2) There are no scale bars in Figure 1A, Figure 6A, Figure 6B, Figure 6D, or Figure 8A.

Response:

We have now added the scale bars.

3) Fig 3B y axis: are the levels of ceramide corrected for protein or Lipid?

Response:

Fig. 3B shows the activity of Asm. It does not show ceramide concentrations. As now stated in section Methods, this figure shows the activity of the enzyme in 30 µl of supernatant. In unstimulated samples (Time point 0), tumor cells and platelets were admixed after lysis. In the stimulated samples, platelets and tumor cells were co-incubated for 20 seconds and lysed. Thus, all samples contained the same amount of tumor cells and platelets. The difference we measured prior or after stimulation is therefore only due to the stimulation of tumor cells by platelets. Samples, in which we measured only platelets or tumor cells, respectively, are indicated with an "-". The samples were corrected for the volume. This is now clearly described in the method.

4) On page 16, the authors mention a role for NK cells in survival of mets and reference data as not shown. This data could add to the paper significantly.

Response:

We now provide our findings with regard to the depletion of natural killer (NK) cells. The results demonstrate that the depletion of NK cells increases metastasis in both wild type and Asm-deficient mice. However, tumor metastasis is still reduced by 77% in Asm-deficient mice, albeit at a higher absolute level. This finding indicates that NK cells and the Asm-mediated adhesion of tumor cells act independently at various levels of tumor metastasis. We assume that NK cells kill most tumor cells before or even after adhesion. However, this killing is not complete. Asm-deficiency reduces tumor cell adhesion by 83%, independent of the actions of NK cells.

5) p10: tumor cell 'arrest' in the lung is not clear terminology; perhaps trapping.

Response:

We have replaced *arrest* with *trapping*.

The blot in Figure 6E is not exposed enough. Please show a longer exposure of this blot.P11; why is melanoma metastasizing to spleen a more 'physiologic model'?

Response:

We repeated the studies with a larger number of cells, and we have now provided improved blots.

The studies using Mt/ret melanoma were performed with mice in which a local melanoma develops and then metastasizes to the spleen. This experimental model better reflects the clinical situation than do intravenous injections of tumor cells. However, because the term *physiological* is not the best, we have now removed this term.

6) P7: what is the evidence that ASM is found in secretory lysosomes?

Response:

We have removed this statement because we have now directly shown that secretory Asm is the relevant isoform in our studies.

Referee #2 (Remarks):

This is an outstanding study showing a role for ASM in tumor metastasis and specifically, a role for ASM secreted from platelet cells. The study is performed at an excellent level of technical competence, and having read it twice, I cannot find any obvious flaws! I only have two minor comments that should be addressed.

The authors suggest that amitriptyline could be used as a therapy. However, this needs more discussion. The authors state that amitriptyline is used at a concentration similar to that used in humans, but do not reference this and nor do they state what disease amitriptyline is used in. Are there off-target effects of amitriptyline? If so, is this a feasible treatment, or do more selective ASM inhibitors need to be developed? Thus, the potential use of amitriptyline needs to be discussed in more detail.

Response:

Also, the authors should expand a little on the mechanism of secretion of ASM from platelets. Would inhibiting ASM secretion be another possible therapeutic approach?

Response:

We have now addressed the mechanisms as follows:

The activity of Asm on the cell surface has been demonstrated by several studies (for instance Schissel et al, 1998a,b; Grassmé et al, 2001, Rotolo et al, 2005; Xu et al, 2012). The results of these studies indicate either that secretory lysosomes containing Asm fuse with the plasma membrane and expose Asm (Grassmé et al, 2001, Rotolo et al, 2005; Herz et al, 2009; Xu et al, 2012).) or that cells secrete Asm, which then binds back to the cell surface (Schissel et al, 1998a,b). This secretory Asm depends on Zn^{2+} for its activity, while lysosomal Asm does not depend on additional Zn^{2+}. We therefore studied the Zn^{2+} dependency of the Asm that is present on tumor cells that have been co-

incubated with platelets. Our findings show that the observed activity of Asm is dependent on Zn^{2+}, a finding indicating that the effects of platelets on B16F10 melanoma cells are mediated by secretory Asm, which is Zn^{2+} dependent.

Additional studies from our laboratory have shown that the secretion of Asm by platelets depends on the expression of P-selectin by platelets. Platelets deficient in P-selectin fail to release Asm upon contact with B16F10 tumor cells, a finding consistent with the markedly reduced metastasis of B16F10 cells in mice deficient in P-selectin. However, because we believe that these findings are beyond the focus of the present manuscript, we only provide this information to the referee, but not for publication.

To validate the finding that Asm is a (preclinical) target for preventing tumor metastasis, we performed studies using both the Asm inhibitor amitriptyline and Asm-heterozygous mice. These mice reflect a pharmacological approach because Asm activity is reduced by only 50%, a level of inhibition that is also achieved clinically with amitriptyline. These results indicate that both amitriptyline and Asm heterozygosity reduce melanoma metastasis by 75 or 83%, respectively, and suggest that Asm may serve as a target for preventing tumor metastasis in patients. These findings are now included in the revised manuscript.

Referee #4 (Comments on Novelty/Model System):

The manuscript describes a novel interaction between host platelets and melanoma cancer cells. Specifically a model is proposed in which platelets release ASM, which promotes formation of ceramide-enriched platforms in the melanoma cells, into which integrins are clustered. This is required for hematogenous metastasis related to enhanced invasive properties of the melanoma cells. There are a number of very well controlled experiments to demonstrate this mechanistic model along with two animal models of metastasis in mice. The evidence for the role of ASM in this process is interesting and provocative and should have significant medical impact if correct e.g. in targeting ASM therapeutically with chemical inhibitors.

However, there are a number of experiments that are required to establish a higher level of proof for the proposed model (see below). Most of the experiments are performed on isolated platelets and melanoma cells in vitro and the observation require direct observation in vivo. In addition, mechanistic evidence for how melanoma cells cause release of ASM from platelets is lacking. Finally, further metabolism of ceramide and exclusion of metabolised products from inducing metastasis are required.

The recommendation is too pursue revision of the manuscript with a favourable view.

Points

i. Demonstrate formation of ceramide enriched domains with clustered integrins in B16F10 and B16F10 in vivo and absence in cells injected into ASM-/- mice; by retrieving cells from mice.

Response:

We labelled B16F10 tumor cells with CFSE, injected the cells into wild type or Asm-deficient mice, withdrew blood 5 min after injection, performed Ficoll gradient centrifugation to remove erythrocytes and to purify tumor cells, fixed the cells, and stained them with a Cy3-labeled anti-ceramide antibody and a Cy5-labeled anti-β1 integrin antibody. The results showed the formation of ceramide-enriched domains in tumor cells (as identified by the intense green labelling of CFSE) that

contain β1 integrin clusters after injection into wild type mice, whereas injecting these labeled tumor cells into Asm-deficient mice did not result in the clustering of ceramide and β1 integrin at the surface of tumor cells. These findings show that the clustering of ceramide and β1 integrin also occurs *in vivo*. The data are provided in Figure 6E.

ii. Provide some details as to the mechanism by which B16F10 and B16F10 cause the release of ASM from platelets

Response:

We have now addressed these mechanisms as follows:

The activity of Asm on the cell surface has been demonstrated by several studies (for instance Schissel et al, 1998a,b; Grassmé et al, 2001, Rotolo et al, 2005; Xu et al, 2012). The results of these studies indicate either that secretory lysosomes containing Asm fuse with the plasma membrane and expose Asm (Grassmé et al, 2001, Rotolo et al, 2005; Herz et al, 2009; Xu et al, 2012) or that cells secrete Asm, which then binds back to the cell surface (Schissel et al, 1998a,b). This secretory Asm depends on Zn^{2+} for its activity, while lysosomal Asm does not depend on additional Zn^{2+}. We therefore studied the Zn^{2+} dependency of the Asm that is present on tumor cells that have been co-incubated with platelets. Our findings show that the observed activity of Asm is dependent on Zn^{2+}, a finding indicating that the effects of platelets on B16F10 melanoma cells are mediated by secretory Asm, which is Zn^{2+} dependent.

Additional studies from our laboratory have shown that the secretion of Asm by platelets depends on the expression of P-selectin by platelets. Platelets deficient in P-selectin fail to release Asm upon contact with B16F10 tumor cells, a finding consistent with the markedly reduced metastasis of B16F10 cells in mice deficient in P-selectin. However, because we believe that these findings are beyond the focus of the present manuscript, we only provide this information to the referee, but not for publication.

iii. Exclude other ASM derived ceramide metabolites involved in metastasis e.g. using ceramide synthase inhibitors, SphK inhibitors and utilise relevant knockouts to establish whether dissemination is affected and whether this is specific to cancer cells or fibroblasts or platelets. For instance, there is evidence from Albinet et al and Ponnusamy et al. for a role for S1P in metastasis and S1P can obviously be derived from ceramide.

Response:

We now measured S1P after co-incubation of B16F10 melanoma cells and wild type platelets. There was no change in S1P secretion (Supplementary Fig. 1 A). In addition, incubating tumor cells with S1P did not restore trapping of tumor cells in the lungs of Asm-deficient mice (Fig. 6H). Incubating tumor cells with a sphingosine kinase inhibitor did not prevent tumor cell arrest and metastasis in wild type mice. Sphingosine kinase inhibitor also did not influence metastasis in Asm-deficient mice (Fig. 6H).

Likewise, treating the tumor cells with PDMP, a glucosyltransferase inhibitor, or with myriocin, an inhibitor of serine palmitoyltransferase and, thereby, of ceramide synthesis, before their injection into mice did not alter lung metastasis (Fig. 6H). Also, treating mice with either PDMP or myriocin before the injection of tumor cells did not alter tumor metastasis (Supplementary Fig. 1).

These findings exclude a significant role of S1P, ceramide synthase, or glucosyltransferase in the mechanism of platelet-tumor (B16F10) interactions.

2nd Editorial Decision 17 February 2015

Thank you for the submission of your revised manuscript to EMBO Molecular Medicine. We have now received the enclosed reports from the referees that were asked to re-assess it. As you will see the reviewers are now globally supportive and I am pleased to inform you that we will be able to accept your manuscript pending the following final amendments:

1) Please abide by Reviewer 1's request to include the indicated important discussion points and his/her request for clarification concerning the mode of delivery of ceramide.

2) As per our Author Guidelines, the description of all reported data that includes statistical testing must state the name of the statistical test used to generate error bars and P values, the number (n) of independent experiments underlying each data point (not replicate measures of one sample), and the actual P value for each test (not merely 'significant' or 'P < 0.05').

3) Please upload individual figure files (i.e. one for each manuscript figure).

4) We are now encouraging the publication of source data, particularly for electrophoretic gels and blots, with the aim of making primary data more accessible and transparent to the reader. Would you be willing to provide a PDF file per figure that contains the original, uncropped and unprocessed scans of all or at least the key gels used in the manuscript (this apart form the specific request of point 4 above)? The PDF files should be labeled with the appropriate figure/panel number, and should have molecular weight markers; further annotation may be useful but is not essential. The PDF files will be published online with the article as supplementary "Source Data" files. If you have any questions regarding this just contact me.

5) Every published paper now includes a 'Synopsis' to further enhance discoverability. Synopses are displayed on the journal webpage and are freely accessible to all readers. They include a short standfirst (to be written by the editor) as well as 2-5 one sentence bullet points that summarise the paper (to be written by the author). Please provide the short list of bullet points that summarise the key NEW findings. The bullet points should be designed to be complementary to the abstract - i.e. not repeat the same text. We encourage inclusion of key acronyms and quantitative information. Please use the passive voice. Please attach these in a separate file or send them by email, we will incorporate them accordingly.

Please submit your revised manuscript as soon as possible so that we can proceed with fromal acceptance.

***** Reviewer's comments *****

Referee #1 (Comments on Novelty/Model System):

this is an impressive study; the major issue remaining is whether acid sphingomyelinase can act on plasma membrane sphingomyelin at neutral pH and in the absence of Zn. the authors can discuss this point

Referee #1 (Remarks):

This is a significantly improved study, and the results with the heterozygous acid sphingomyelinase (Asm) mouse are extremely important.

There are a couple of remaining issues:
1. The authors claim that aSMase is active once is released, While the group of Tabas has shown Asm can act on sphingomyelin in lipoproteins, the possible effect of secreted Asm on cell membrane sphingomyelin has not been rigorously establishes. This issue is further compounded by the authors' own results confirming the need for Zn for activity of this enzyme. This point should be at least discussed
2. The sphingolipid profile is limited to ceramide. There should be some discussion on whether the effects are due to ceramide or a downstream metabolite.

3. How was c16 ceramide delivered in the cell studies?

Referee #4 (Comments on Novelty/Model System):

The study is novel and is likely to have high impact in the biomedical area

Referee #4 (Remarks):

All of the comments have been addressed by the authors

2nd Revision - authors' response 01 March 2015

Thank you very much for the positive review of our manuscript. As outlined below, we have addressed all remaining issues in detail.

We hope the revised manuscript is acceptable for EMBO Mol. Med.

If I can be of any further assistance, please let me know anytime.

1) Please abide by Reviewer 1's request to include the indicated important discussion points and his/her request for clarification concerning the mode of delivery of ceramide.

Response: Please see below the answers to the referees.

2) As per our Author Guidelines, the description of all reported data that includes statistical testing must state the name of the statistical test used to generate error bars and P values, the number (n) of independent experiments underlying each data point (not replicate measures of one sample), and the actual P value for each test (not merely 'significant' or 'P < 0.05').

Response: We now name the test and give the exact p-values. We already gave all n-numbers.

3) Please upload individual figure files (i.e. one for each manuscript figure).

Response: We now ordered the figures as one file per figure.

4) We are now encouraging the publication of source data, particularly for electrophoretic gels and blots, with the aim of making primary data more accessible and transparent to the reader. Would you be willing to provide a PDF file per figure that contains the original, uncropped and unprocessed scans of all or at least the key gels used in the manuscript (this apart form the specific request of point 4 above)? The PDF files should be labeled with the appropriate figure/panel number, and should have molecular weight markers; further annotation may be useful but is not essential. The PDF files will be published online with the article as supplementary "Source Data" files. If you have any questions regarding this just contact me.

Response: The PDF files of the entire/raw original blots are provided.

5) Every published paper now includes a 'Synopsis' to further enhance discoverability. Synopses are displayed on the journal webpage and are freely accessible to all readers. They include a short standfirst (to be written by the editor) as well as 2-5 one sentence bullet points that summarise the paper (to be written by the author). Please provide the short list of bullet points that summarise the key NEW findings. The bullet points should be designed to be complementary to the abstract - i.e. not repeat the same text. We encourage inclusion of key acronyms and quantitative information.

Please use the passive voice. Please attach these in a separate file or send them by email, we will incorporate them accordingly.

Response: We now added a synopsis and the section „The paper explained"

***** Reviewer's comments *****

Referee #1 (Comments on Novelty/Model System):

this is an impressive study; the major issue remaining is whether acid sphingomyelinase can act on plasma membrane sphingomyelin at neutral pH and in the absence of Zn. the authors can discuss this point

Response: It is important to note that not only the pH value, but also the lipid environment is critical for the activity of the acid sphingomyelinase as previously shown by Schissel et al. and recently studied in detail by Oninla et al. Further, blood plasma contains sufficient Zn^{2+} concentrations that allow the activity of the enzyme (Spence et al., 1989). Please see below for a detailed response.

Referee #1 (Remarks):

This is a significantly improved study, and the results with the heterozygous acid sphingomyelinase (Asm) mouse are extremely important.

There are a couple of remaining issues:

1. The authors claim that aSMase is active once is released, While the group of Tabas has shown Asm can act on sphingomyelin in lipoproteins, the possible effect of secreted Asm on cell membrane sphingomyelin has not been rigorously establishes. This issue is further compounded by the authors' own results confirming the need for Zn for activity of this enzyme. This point should be at least discussed.

Response: We now extended the discussion that reads as following:

Zn^{2+} is a nutrient and is present in the blood plasma at concentrations that allow activity of the secretory Asm (Spence MW, Byers DM, Palmer FBSC, Cook HW (1989) A new Zn^{2+}-stimulated sphingomyelinase in fetal bovine serum. J Biol Chem 264:5358-5363). Secretory Asm has been previously shown to be active even at a neutral pH (Schissel et al, 1998a,b). Furthermore, pH determines the K_m but not the V_{max} of enzyme activity (Schissel et al, 1998a,b). The K_m value of sphingomyelin depends on the lipid environment and enables Asm to be active at a more neutral pH. A recent study indicated that the activity of the acid sphingomyelinase is regulated by anionic plasma membrane phospholipids (Oninla VO, Breiden B, Babalola JO, Sandhoff K (2014) Acid sphingomyelinase activity is regulated by membrane lipids and facilitates cholesterol transfer by NPC. J Lipid Res 55: 2606-2619). In particular phosphatidylglycerol and phosphatidic acid increase Asm activity. Asm-activity is also increased by other lipids in the plasma membrane, partly generated by the activity of rhe Asm itself. These lipids are ceramide, diacylglycerol and free fatty acids. Finally, further studies by our group indicate that H^+-ATPase clusters in ceramide-enriched membrane domains on the surface of B16F10 cells after these cells were co-incubated with platelets (Supplementary Figure 2), a finding suggesting an acidification of these domains, as previously shown by Xu et al. (2012). Secretory Asm has been previously show to bind to cell surfaces (Dhami R, Schuchman EH (2004) Mannose 6-phospohate receptor-mediated uptake is defective in acid sphingomyleinase-deficient macrophages. J Biol Chem 279: 1526-1532; Schissel SL, Keesler GA, Schuchman EH, Williams KJ, Tabas I (1998) The cellular trafficking and zinc dependence of secretory and lysosomal sphingomyelinase, two products of the acid sphingomyelinase gene. J Biol

Chem 273: 18250-18259) allowing the enzyme to be active on the cell surface. Although the exact mechanisms how that enzyme binds to the cell surface remain to be determined, the lipid composition of the plasma membrane, the activity of the secretory Asm at higher pH values, the presence of H^+-ATPase in ceramide-enriched domains potentially acidifying surface microdomains and the concentrations of Zn^{2+} in the plasma allow activity of the Asm on the cell surface.

2. The sphingolipid profile is limited to ceramide. There should be some discussion on whether the effects are due to ceramide or a downstream metabolite.

Response: We now added the following section:

Our studies suggest a primary role of Asm-released ceramide in tumor cell metastasis, although they do not exclude a role of other sphingolipids. However, treatment of the tumor cells with inhibitors of sphingosine kinases, the ceramide synthesis pathway, DL-threo-1-phenyl-2-decanoylamino-3-morpholino-1-propanol (PDMP), glucosyltransferases, or pre-treatment with S1P are without effect on tumor cell metastasis. Further, S1P concentrations did not change after co-incubation of tumor cells with platelets. These data exclude a role of glucosylceramides and S1P in the observed metastasis. In addition, we show that an Asm-dependent activation of integrins is required for tumor metastasis. Studies from our group excluded clustering of β1 integrins upon treatment with sphingosine (not shown). However, it cannot be excluded that ceramide is converted to ceramide 1-phosphate that may, for instance via phospholipase A_2 activation, contribute to tumor metastasis.

3. How was c16 ceramide delivered in the cell studies?

Response: C16 Ceramide was delivered in octylgluocopyranoside (10% stock, final concentration: 0.01%). The controls are with the same concentration of OGP without ceramide. This is now more clearly stated in the method section.
