# Supplementary material for: Regulation of hematogenous tumor metastasis by acid sphingomyelinase
Source: EMBO Mol Med. 2015 Apr 7;7(6):714–34. doi: 10.15252/emmm.201404571 (PMC4459814; doi:10.15252/emmm.201404571)
Supplement: Supplementary file 3 [file emmm0007-0714-sd3.pdf]

Blots: Raw data, unprocessed

Figure 6 A: left blot

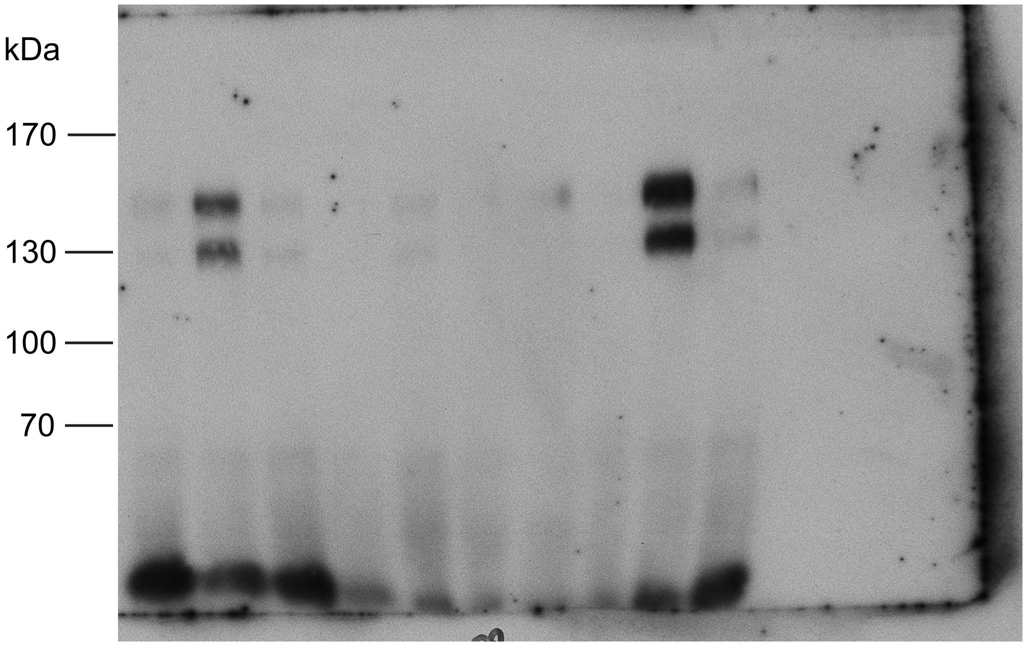

Figure 6 A: left blot loading control

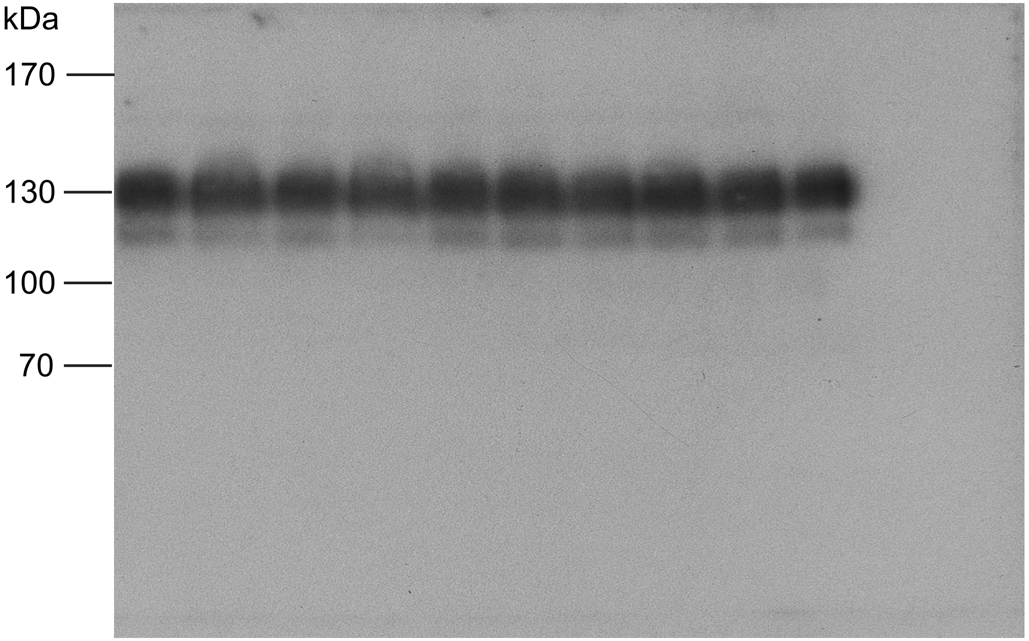

Figure 6 A: right blot

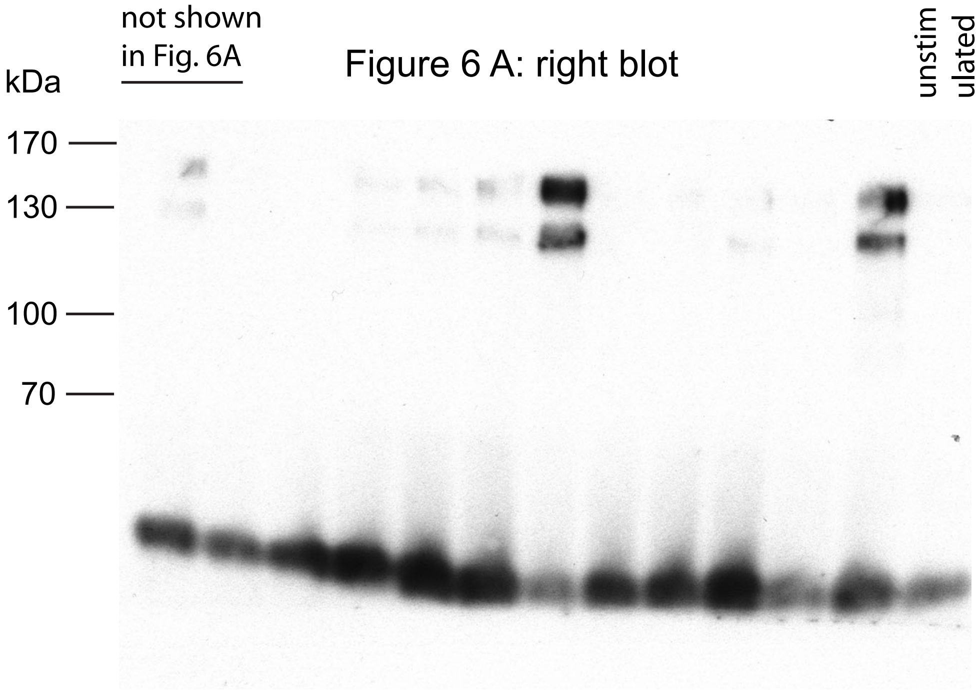

Figure 6 A: right blot loading control

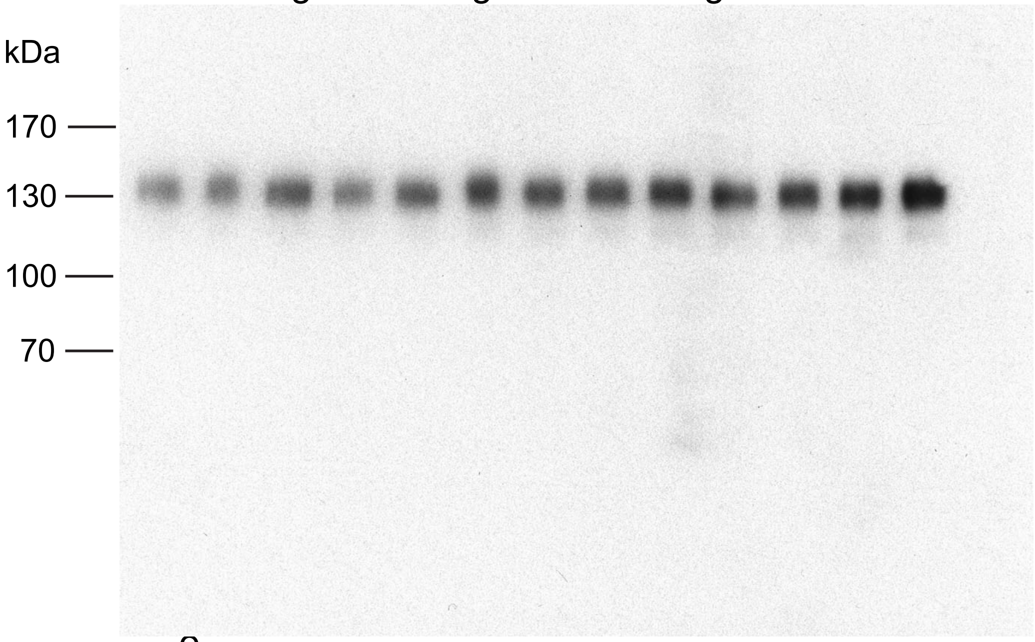

Please note: The blot in the right panel of Fig. 6A was rotated by 180° to start the unstimulated sample on the left hand side. The last left two lanes (here on the right hand side as indicated are a 2nd control Ipt and an untreated sample and are not shown in Fig. 6A.
